# Computational time-dependent acoustic scattering in a rectangular membrane–porous waveguide

**DOI:** 10.1371/journal.pone.0358579

**Published:** 2026-09-30

**Authors:** Muna Elsadig, Muhammad Safdar, Hala H. Taha, Muhammad Afzal

**Affiliations:** 1 Department of Information Systems, College of Computer and Information Sciences, Princess Nourah bint Abdulrahman University, Riyadh, Saudi Arabia; 2 Department of Mathematics, Quaid-i-Azam University, Islamabad, Pakistan; 3 Department of Mathematical Sciences, College of Science, Princess Nourah bint Abdulrahman University, Riyadh, Saudi Arabia; 4 Department of Mathematics and Natural Sciences, Gulf University for Science and Technology, Kuwait; COMSATS University Islamabad, PAKISTAN

## Abstract

A semi-analytical, computational framework is developed for the time-dependent propagation, scattering and dissipative decay of a broadband acoustic pulse in a two-dimensional rectangular waveguide whose central segment carries a porous material core bracketed by two clamped flexible membrane strips. The transient treatment follows the wavepacket–Fourier approach. About the centre line the field splits into symmetric and anti-symmetric subproblems; the duct and porous segments are expanded on rigid-wall cosine modes and each membrane on a clamped sine Galerkin basis, whose projection yields a closed-form coupling matrix. The block linear system is validated by point-wise reconstruction of pressure and velocity continuity at every matching plane, by a truncation convergence study, and by two independent energy checks: the absorbed power fraction is recomputed from a dissipation audit of the power fluxes entering and leaving the porous core, and the lossless limit of the porous model is shown to return the incident power in full. The time-dependent field is recovered as a single Gaussian-weighted matrix product over a precomputed wavenumber grid, so that each snapshot costs one matrix–vector product. Numerical experiments delineate transmission, mixed and reflection regimes, locate the membrane–cavity hybrid resonances of maximum absorption, and visualise the transfer of pulse energy to the membranes and porous filler during transit.

## 1 Introduction

The control of broadband acoustic disturbances inside ducted systems is a recurrent design problem in heating, ventilation and air-conditioning networks, in exhaust mufflers, in compact loudspeaker enclosures, and in the architectural and aerospace treatment of partitions, building components and aircraft nacelles. Two complementary engineering principles dominate the analytical response. The first is to break the duct with a dissipative porous insert whose fibrous or microcellular skeleton converts acoustic energy into heat through viscothermal losses. The second is to deploy thin flexible elements – membranes, micro-perforated panels, compliant plates – that couple the acoustic field to a structural resonance and so introduce a reactive impedance jump that selectively reflects narrow bands of frequency. Hybrid configurations that combine both elements – a porous core bracketed by flexible terminations – exploit the complementary strengths of the two strategies and yield filters whose reflection, transmission and absorption fractions can be tailored over a broad frequency band.

A substantial literature has grown around the semi-analytical modelling of such hybrid devices. Expansion-chamber silencers were analysed by Selamet and Radavich [1], topology optimisation of muffler partitions by Lee and Kim [2], the interaction of micro-perforated panels with surrounding fluid layers by Maxit et al. [3] and by Yu et al. [4,5], and double-layer and subdivided-cavity micro-perforated absorbers by Zhang and Gu [6] and by Toyoda and Takahashi [7]. Single-layer liners backed by porous honeycombs were measured by Hillereau et al. [8]. More recent work has addressed trifurcated and branched configurations [9], parallel micro-perforated sub-absorbers [10,11], the continuity of velocity at perforation boundaries [12], and building-acoustic applications of flexible-absorbent ducts [13]. Dissipative silencers lined with poroelastic materials and containing mean flow have been treated by Nennig et al. [14] and benchmarked against numerical methods by Kirby [15].

Within this body of work the analytical line most directly relevant to the present paper combines the mode-matching method (MMM) with a Galerkin treatment of the membrane discontinuity. Two-dimensional acoustic propagation in a duct bounded by elastic membranes was analysed by Nawaz et al. [16]; acoustic propagation in three-dimensional rectangular ducts with flexible walls and porous linings was treated by Lawrie [17], and Lawrie and Afzal introduced the tailored Galerkin technique for waveguides with height discontinuities bridged by membranes [18]. A sequence of extensions has since refined the methodology to wave-bearing cavities [19], to partitioned membrane-bounded cavities with material contrast [20], to silencing membrane cavities with different edge conditions [21], to flexural attenuation with absorbent linings and different edge conditions [22], to flexible waveguides with absorbent material along flanged junctions [23], to coaxial lined chambers with membrane discs [24], to sound-source modelling with structural variations and sound-absorbent materials [25], to bifurcated flexible shells [26] and dynamical surfaces tailored in a flexible shell [27], and to the optimisation of acoustic liner conditions with flexible components [28]. A cylindrical mode-filter combining a porous central cavity with two clamped flexible membrane discs has likewise been reported by Alahmadi et al. [29].

Time-dependent visualisation of the same kind of mode-matching problem has progressed in parallel. Rather than reporting a single frequency response, the frequency-domain solution is evaluated on a dense wavenumber grid and recombined against a localised wavepacket envelope through a Fourier integral. The methodology was brought to the rectangular-waveguide-with-corner setting by Aldosri and Meylan [30], who emphasised both the qualitative insight a moving snapshot reveals and the algorithmic observation that the spatial matrix can be cached so that each new time instant costs only one matrix–vector product. In the frequency domain, the filtering of higher-order modes by dissipative screens in a rigid duct has recently been analysed by Lawrie and Afzal [31], who considered an inlet–outlet system carrying a finite-length section fitted with resistive wire-mesh perforates and characterised the configurations of optimum higher-mode attenuation in terms of the coalescence of duct eigenmodes at exceptional points, the plane wave passing essentially unimpeded.

It is worth stating precisely what separates the present study from its closest antecedents. In the corner-scatterer study of Aldosri and Meylan [30] – the time-domain antecedent, which already treats a rectangular geometry with the same wavepacket–Fourier reconstruction – the scattering surfaces are rigid, and in the wire-mesh filtering study of Lawrie and Afzal [31] the dissipative screens are purely resistive: in both cases the interface conditions are algebraic relations between the acoustic traces on either side, so each frequency solve reduces to mode matching alone, with no structural unknowns. Here, by contrast, the terminations of the dissipative core are elastic membranes that carry their own dynamics: each frequency solve is a coupled fluid–structure problem in which the membrane displacement is an additional unknown field governed by its own differential equation with clamped edges. The methodological contribution is the treatment of this coupling in the Cartesian rectangular setting: the membrane displacement is expanded on a sine basis that satisfies both clamped edges identically, the projection of the rigid-duct cosine modes onto that basis is obtained in closed form, and the resulting Galerkin systems – one per parity subproblem – absorb the porous-core impedance through an exact two-term elimination, so that the only linear system solved has the dimension of the membrane basis. None of these ingredients appears in the rigid-scatterer or resistive-screen studies cited above, and their combination with the wavepacket–Fourier reconstruction, together with a parity selection rule that governs which transverse modes a symmetric filter can pass, is to the best of our knowledge new.

Beyond this methodological gap, two further gaps motivate the study. First, the time-domain analysis cited above concentrates on rigid stepped or right-angle scatterers [30], and the dissipative screens of the frequency-domain filtering study [31] are purely resistive; the broadband transient behaviour of a *composite* reactive (membrane) and dissipative (porous bulk) filter remains, to the best of our knowledge, undocumented. Second, the behaviour of a transient pulse near the membrane and cavity resonances is qualitatively different from the steady-state energy curves usually plotted – a transient frame can make absorption and reflection visible as ringing rather than as a single number.

The present article addresses these gaps by formulating, solving, validating and reporting the time-dependent acoustic scattering problem for a rectangular waveguide that carries two clamped flexible membrane strips bracketing a porous central cavity. The solution is built from a symmetric and an anti-symmetric subproblem, a Galerkin projection of the membrane equation onto a clamped basis, and the energy bookkeeping of reflected, transmitted and absorbed power, all derived directly for the Cartesian rectangular geometry. Time-dependence is introduced through the Gaussian-wavepacket reconstruction of [30], specialised to the present hybrid filter, so that the spatial coefficient matrix is computed once and re-used at every time instant. Validation precedes all reported numbers and figures: a point-wise interface reconstruction, a truncation convergence study, and an independent energy audit – a dissipation integral over the porous core together with a lossless-limit check – must all pass before the driver writes a single result file.

The remainder of the paper is organised as follows. The physical model, governing equations, boundary conditions and membrane conditions are stated first, followed by the symmetric and anti-symmetric mode-matching solution and the matrix system. The time-dependent reconstruction and the power identities – including the independent energy audit – are then described. The numerical results are reported in a single integrated section that opens with the validation of the truncated solution, then exposes the role of higher-order acoustic modes, the modal-filtering response to individual higher-order incident modes, the parametric heatmaps of the absorbed fraction against the porous half-length, the membrane tension and the duct half-width, the steady-state spatial field over the full geometry, the time-domain snapshots of the broadband wavepacket, and finally the transient energy stored in the porous core during transit. A concluding section summarises the findings, and S1 Appendix records the intermediate algebra connecting the membrane equation to the Galerkin systems.

## 2 Physical model and governing equations

The geometry is shown schematically in Fig 1. A two-dimensional rectangular waveguide of half-width *a* extends along the axial coordinate x∈(−∞,∞), with rigid walls at y=±a. Two clamped flexible membrane strips, each spanning the duct cross-section, are placed at x=−2L and *x* = +2*L*. Between the membrane strips the waveguide is partitioned by two stationary impedance interfaces at x=−L and *x* = +*L* into three axial segments: two air-filled side cavities adjacent to each membrane and a central segment filled with a fibrous porous medium modelled by the Delany–Bazley empirical relations [32]. The five regions used in the modal analysis are


$$\begin{array}{c} \begin{array}{cc} \Omega_{1}:=\{(x,y):x<-2L,\;-a<y<a\}, & \text{(inlet)}\\ \Omega_{2}:=\{(x,y):-2L<x<-L,\;-a<y<a\}, & \text{(left air cavity)}\\ \Omega_{3}:=\{(x,y):-L<x<L,\;-a<y<a\}, & \text{(porous core)}\\ \Omega_{4}:=\{(x,y):L<x<2L,\;-a<y<a\}, & \text{(right air cavity)}\\ \Omega_{5}:=\{(x,y):x>2L,\;-a<y<a\}, & \text{(outlet).} \end{array} \end{array}$$

**Fig 1:**
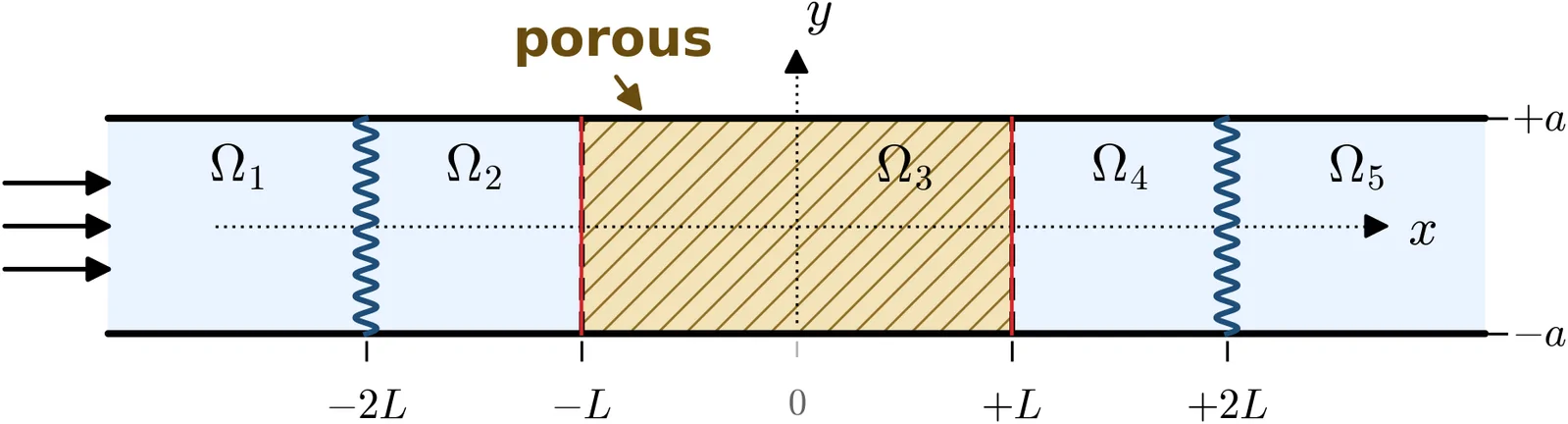
Geometry of the rectangular waveguide. Air-filled regions are shaded light blue and the porous core is shaded tan. The solid black lines at y=±a are the rigid duct walls; the wavy blue profiles at x=±2L are the clamped flexible membrane strips; the red dashed lines at x=±L are the stationary air-porous interfaces. Dotted red lines mark the Cartesian axes through the origin, and tick marks at x=±L,±2L and y=±a identify the five region boundaries. The black arrow indicates the direction of the incident wave.

Let φ(x,y,t) denote the acoustic velocity potential. In every region the field satisfies the wave equation


$$\begin{array}{c} \nabla^{2}\varphi -\frac{1}{c_{j}^{2}}\;\partial_{t}^{2}\varphi =0, \end{array}$$ (1)

with $c_{j}$ equal to the (real) sound speed *c* in the air-filled regions $\Omega_{1},\Omega_{2},\Omega_{4},\Omega_{5}$ and an effective complex sound speed c/Γ in the porous core $\Omega_{3}$, where Γ is the dimensionless Delany–Bazley propagation constant. We pass to the time-harmonic representation $\varphi (x,y,t)=Re\{\phi (x,y)e^{-i\omega t}\}$ for the frequency-domain analysis and recover φ later from a wavepacket integral over ω. The complex potential then satisfies


$$\begin{array}{c} (\nabla^{2}+k^{2})\;\phi_{j}(x,y)=0,\;j\in \{1,2,4,5\},\;k=\omega /c, \end{array}$$ (2)

in the air-filled regions, and


$$\begin{array}{c} (\nabla^{2}+(k\Gamma {)}^{2})\;\phi_{3}(x,y)=0 \end{array}$$ (3)

in the porous core. The Delany–Bazley constants are


$$\begin{array}{c} \Gamma =1+ia_{1}\xi^{a_{2}}+a_{3}\xi^{a_{4}},\;\tau =\Gamma \;[1+a_{5}\xi^{a_{6}}-ia_{7}\xi^{a_{8}}],\;\xi =f\rho /\sigma , \end{array}$$ (4)

in which f=ω/(2π) is the frequency, ρ is the air density, σ is the flow resistivity of the fibrous filler – so that ξ is the dimensionless Delany–Bazley frequency parameter [32] – and τ is the complex effective-density ratio that controls the pressure impedance of the porous filler relative to air. For fibrous media the flow resistivity is conventionally estimated from the bulk density $\rho_{b}$ of the fibrous skeleton – not from the air density – through the empirical power law $\sigma =A_{1}\rho_{b}^{A_{2}}$, with material constants *A*_1_ and *A*_2_ obtained from the experimental characterisations collected by Kirby and Cummings [33]. The numerical values of *a*_1_–*a*_8_, *A*_1_, *A*_2_, the resulting value of σ actually used, and the realised range of ξ are all reported in the results section, together with a discussion of the validity window of the correlations.

The rigid duct walls give Neumann conditions


$$\begin{array}{c} \partial_{y}\phi_{j}(x,\pm a)=0,\;\text{for every region }j. \end{array}$$ (5)

At each stationary porous interface, pressure continuity contributes the complex factor τ – so the velocity potentials in air and in the porous core are related by


$$\begin{array}{c} \phi_{2}(-L,y)=\tau \;\phi_{3}(-L,y),\;\phi_{4}(+L,y)=\tau \;\phi_{3}(+L,y), \end{array}$$ (6)

while the normal component of the particle velocity is continuous across each interface,


$$\begin{array}{c} \partial_{x}\phi_{2}(-L,y)=\partial_{x}\phi_{3}(-L,y),\;\partial_{x}\phi_{4}(+L,y)=\partial_{x}\phi_{3}(+L,y). \end{array}$$ (7)

The transverse displacement *U*_1_(*y*) of the left membrane strip at x=−2L, measured positive along +*x* (toward the chamber interior), satisfies the clamped-edge boundary value problem


$$\begin{array}{c} \frac{d^{2}U_{1}}{dy^{2}}+k_{p}^{2}\;U_{1}=\frac{1}{T}(p_{2}-p_{1}){|}_{x=-2L},\;U_{1}(\pm a)=0, \end{array}$$ (8)

with $k_{p}=\omega /c_{m}$ and $c_{m}=\sqrt{T/\rho_{m}}$ the membrane wave speed obtained from the membrane tension *T* and surface mass density $\rho_{m}$. The fluid pressure jump across the membrane is converted to velocity potential through $p_{j}=-i\omega \rho \;\phi_{j}$ so the membrane forcing reads $\left(p_{2}-p_{1})=-i\omega \rho \;(\phi_{2}-\phi_{1}\right)$. The corresponding fluid normal-velocity continuity conditions across the left membrane are


$$\begin{array}{c} \partial_{x}\phi_{1}(-2L,y)=-i\omega \;U_{1}(y),\;\partial_{x}\phi_{2}(-2L,y)=-i\omega \;U_{1}(y). \end{array}$$ (9)

Because Eq (8) is second order in the transverse coordinate, only the edge displacement can be prescribed; the term “clamped” here therefore coincides with fixed (zero-displacement) edges, and no edge-slope condition arises – in contrast to fourth-order plate models, in which a clamped edge additionally constrains the rotation.

The right membrane at *x* = +2*L* obeys the mirror image of Eqs (8) and (9). With the displacement *U*_2_(*y*) measured positive along −x – that is, again toward the chamber interior, so that the two membrane problems are exact mirror images of one another – the boundary value problem is


$$\begin{array}{c} \frac{d^{2}U_{2}}{dy^{2}}+k_{p}^{2}\;U_{2}=\frac{1}{T}(p_{4}-p_{5}){|}_{x=+2L},\;U_{2}(\pm a)=0, \end{array}$$ (10)

with the fluid coupling


$$\begin{array}{c} \partial_{x}\phi_{4}(+2L,y)=+i\omega \;U_{2}(y),\;\partial_{x}\phi_{5}(+2L,y)=+i\omega \;U_{2}(y). \end{array}$$ (11)

The orientation convention is thus uniform: on each membrane the driving pressure jump is the interior-side minus the exterior-side pressure, and the displacement is positive when the membrane bulges toward the porous core. In the mirror decomposition introduced below, the conditions of Eqs (10) and (11) are satisfied identically once the left-membrane conditions hold in both parity subproblems; S1 Appendix makes this explicit.

The model neglects mean flow, membrane damping and membrane bending stiffness. The neglect of mean flow restricts attention to the low-Mach-number regime typical of ventilation and building-services ducts: dissipative-silencer analyses that include mean flow [14,15] find convective corrections to the axial wavenumbers of the order of the Mach number, so the present results should be read as the leading-order description for Mach numbers up to a few percent. Membrane damping, if included, would round and lower the sharp hybrid-resonance absorption peaks reported below without displacing them appreciably; and bending stiffness is negligible for the thin, tension-dominated films considered here, for which the tension term dominates the flexural rigidity at the transverse wavelengths resolved by the Galerkin basis.

## 3 Mode-matching solution

The duct cross-section eigenfunctions for the air-filled regions are


$$\begin{array}{c} Y_{n}(y)=\cos(\alpha_{n}(y+a)),\;\alpha_{n}=\frac{n\pi }{2a},\;n=0,1,2,..., \end{array}$$ (12)

which automatically satisfy the rigid-wall conditions of Eq (5), and are orthogonal over (−a,a) with norms $X_{n}=\int_{-a}^{a}Y_{n}(y{)}^{2}\;dy=2a$ if *n* = 0 and *a* otherwise. The associated axial wavenumbers in the air-filled regions are $\eta_{n}=\sqrt{k^{2}-\alpha_{n}^{2}}$ and in the porous core $s_{n}=\sqrt{(k\Gamma {)}^{2}-\alpha_{n}^{2}}$; the principal branch is taken so that evanescent modes decay at +∞. The clamped membrane strips are represented in the Galerkin basis


$$\begin{array}{c} \psi_{m}(y)=\sin(\lambda_{m}(y+a)),\;\lambda_{m}=\frac{m\pi }{2a},\;m=1,2,..., \end{array}$$ (13)

which vanish identically at y=±a and are orthogonal with $\int_{-a}^{a}\psi_{m}\psi_{n}\;dy=a\;\delta_{mn}$.

The projection of the cosine modes onto the sine basis is the closed form


$$\begin{array}{c} R_{mn}=\int_{-a}^{a}\sin(\lambda_{m}(y+a))\cos(\alpha_{n}(y+a))\;dy=\left\{ \begin{array}{cc} \frac{4am}{\pi (m^{2}-n^{2})} & \text{if }m+n\text{odd},\\ 0 & \text{otherwise}. \end{array}\right. \end{array}$$ (14)

The structure is symmetric about *x* = 0, so the standard mirror decomposition splits the original problem into a symmetric subproblem (superscript *s*) with a virtual rigid plane at *x* = 0 and an anti-symmetric subproblem (superscript *a*) with a virtual soft plane at *x* = 0. In each subproblem, only the half-line *x* < 0 is solved; the field on *x* > 0 is recovered by mirroring. The symmetric expansions read explicitly


$$\begin{array}{cc} \phi_{1}^{s} & =e^{ik(x+2L)}+\sum_{n=0}^{\infty }A_{n}^{s}\;Y_{n}(y)\;e^{-i\eta_{n}(x+2L)}, \end{array}$$ (15)

$$\begin{array}{cc} \phi_{2}^{s} & =\sum_{n=0}^{\infty }[B_{n}^{s}\;e^{i\eta_{n}(x+L)}+C_{n}^{s}\;e^{-i\eta_{n}(x+L)}]\;Y_{n}(y), \end{array}$$ (16)

$$\begin{array}{cc} \phi_{3}^{s} & =2\sum_{n=0}^{\infty }D_{n}^{s}\;\cos(s_{n}x)\;Y_{n}(y). \end{array}$$ (17)

The anti-symmetric counterparts have the same form except that the porous expansion is replaced by $\phi_{3}^{a}=2i\sum_{n}D_{n}^{a}\;\sin(s_{n}x)\;Y_{n}(y)$.

Pressure and velocity continuity at x=−L – relations (6) and (7) – give $B_{m}^{s}+C_{m}^{s}=2\tau D_{m}^{s}\cos(s_{m}L)$ and $B_{m}^{s}-C_{m}^{s}=-2i(s_{m}/\eta_{m})D_{m}^{s}\sin(s_{m}L)$; combining,


$$\begin{array}{c} B_{m}^{s}e^{-i\eta_{m}L}+C_{m}^{s}e^{i\eta_{m}L}=2F_{m}\;D_{m}^{s},\;B_{m}^{s}e^{-i\eta_{m}L}-C_{m}^{s}e^{i\eta_{m}L}=-2i\;G_{m}\;D_{m}^{s}, \end{array}$$ (18)

where


$$\begin{array}{cc} F_{m} & =\tau \cos(s_{m}L)\cos(\eta_{m}L)-\frac{s_{m}}{\eta_{m}}\sin(s_{m}L)\sin(\eta_{m}L),\\ G_{m} & =\tau \cos(s_{m}L)\sin(\eta_{m}L)+\frac{s_{m}}{\eta_{m}}\sin(s_{m}L)\cos(\eta_{m}L). \end{array}$$ (19)

Substituting Eqs (15)–(17) into the membrane equation (8) and projecting onto $\psi_{n}(y)$ yields


$$\begin{array}{c} a(k_{p}^{2}-\lambda_{n}^{2})\;w_{n}^{s}-i\omega^{2}\rho /T\sum_{p}w_{p}^{s}\sum_{m}R_{mn}R_{mp}\;\frac{iF_{m}+G_{m}}{\eta_{m}X_{m}G_{m}}=\frac{2i\omega \rho }{T}\;R_{n0}, \end{array}$$ (20)

in which the Galerkin coefficients $w_{n}^{s}$ define the membrane displacement $U^{s}(y)=\sum_{m}w_{m}^{s}\;\psi_{m}(y)$. The corresponding anti-symmetric system is


$$\begin{array}{c} a(k_{p}^{2}-\lambda_{n}^{2})\;w_{n}^{a}+i\omega^{2}\rho /T\sum_{p}w_{p}^{a}\sum_{m}R_{mn}R_{mp}\;\frac{i{\tilde{G}}_{m}-{\tilde{F}}_{m}}{\eta_{m}X_{m}{\tilde{F}}_{m}}=\frac{2i\omega \rho }{T}\;R_{n0}, \end{array}$$ (21)

with


$$\begin{array}{cc} {\tilde{F}}_{m} & =\frac{s_{m}}{\eta_{m}}\cos(s_{m}L)\cos(\eta_{m}L)-\tau \sin(s_{m}L)\sin(\eta_{m}L),\\ {\tilde{G}}_{m} & =\tau \sin(s_{m}L)\cos(\eta_{m}L)+\frac{s_{m}}{\eta_{m}}\cos(s_{m}L)\sin(\eta_{m}L). \end{array}$$ (22)

The intermediate algebra that leads from Eq (8) to the two Galerkin systems – including the elimination of the cavity and porous amplitudes, the recovery formulas for all modal coefficients, and the sign conventions – is collected step by step in S1 Appendix, so that every coefficient in Eqs (20) and (21) can be verified independently.

Once each subproblem is solved for its Galerkin vector, the reflected and transmitted modal amplitudes follow from the mirror combination


$$\begin{array}{c} R_{n}=\frac{1}{2}(A_{n}^{s}+A_{n}^{a}),\;T_{n}=\frac{1}{2}(A_{n}^{s}-A_{n}^{a}), \end{array}$$ (23)

which combine the symmetric and anti-symmetric inlet-mode amplitudes into the physical reflected and transmitted modal coefficients.

## 4 Time-dependent reconstruction

For a broadband incident pulse with spectral envelope $\hat{f}(k)$, the time-dependent total field is recovered from the family of frequency-domain solutions through the Fourier integral


$$\begin{array}{c} \varphi (x,y,t)=Re\{\int_{0}^{\infty }\hat{f}(k)\;\phi (x,y;k)\;e^{-ickt}\;dk\}. \end{array}$$ (24)

The spectral envelope is taken to be a Gaussian wavepacket centred at the carrier wavenumber *k*_0_ (rad/m) with bandwidth $\sigma_{k}$ (rad/m),


$$\begin{array}{c} \hat{f}(k)=\frac{1}{\sigma_{k}\sqrt{\pi }}\;\exp\;[-((k-k_{0})/\sigma_{k}{)}^{2}], \end{array}$$ (25)

which yields a localised spatial envelope and an exact Fourier transform. Because the incident wave in Eq (15) carries the phase factor $e^{ik(x+2L)}$, the envelope centre of the incident packet travels along x=ct−2L: the packet centre crosses the left membrane plane x=−2L at *t* = 0, negative times show the approach through the inlet, and positive times show the transit and *t*he ring-down. All snapshot times quoted below refer to this origin. The integral of Eq (24) is discretised on the uniform grid $\{k_{i}{\}}_{i=1}^{N_{k}}$ with midpoint-rule weights $w_{i}$ and expressed as a matrix product,


$$\begin{array}{c} \varphi ({\vec{x}}_{j},t)\approx Re\{\sum_{i=1}^{N_{k}}M_{ji}\;e^{-ick_{i}t}\},\;M_{ji}:=\phi ({\vec{x}}_{j};k_{i})\;\hat{f}(k_{i})\;w_{i}. \end{array}$$ (26)

The spatial matrix *M* is assembled once and re-used at every time instant, so each new snapshot costs one matrix–vector product. Two wavenumber grids are used in the numerical experiments below. The field snapshots use $N_{k}=40$ points on the interval $\left[k_{0}-2.5,\;k_{0}+2.5\right]$ rad/m with bandwidth $\sigma_{k}=1.0$ rad/m, i.e., a span of $\pm 2.5\;\sigma_{k}$ about the carrier *k*_0_ = 9.15 rad/m of the reference frequency; the transient-energy studies use $N_{k}=80$ points on $\left[k_{0}-3.5\sigma_{k},\;k_{0}+3.5\sigma_{k}\right]$ with $\sigma_{k}=2.5$ rad/m. Restricting the integral of Eq (24) to *k* > 0 is immaterial for every case reported: the Gaussian mass of Eq (25) below *k* = 0 is $\frac{1}{2}\;erfc(k_{0}/\sigma_{k})$, which is at most $9.5\times 10^{-4}$ for the lowest carrier–bandwidth combination used (carrier frequency 300 Hz with $\sigma_{k}=2.5$ rad/m) and below $1.2\times 10^{-7}$ in every other case.

## 5 Power identities and independent energy audit

The reflected and transmitted modal amplitudes $R_{n}$ and $T_{n}$ from Eq (23) carry the unit-incident-flux normalised reflected and transmitted power fractions


$$\begin{array}{c} E_{r}:=\frac{1}{2ka}\;\;\sum_{n:\;\eta_{n}\in \mathbb{R}}\;|R_{n}{|}^{2}\;\eta_{n}\;X_{n},\;E_{t}:=\frac{1}{2ka}\;\;\sum_{n:\;\eta_{n}\in \mathbb{R}}\;|T_{n}{|}^{2}\;\eta_{n}\;X_{n},\;E_{abs}:=1-E_{r}-E_{t}, \end{array}$$ (27)

in which only cut-on modes – those with real $\eta_{n}$ – contribute to the radiated balance and $X_{n}=\int_{-a}^{a}Y_{n}(y{)}^{2}\;dy$ is the non-conjugated norm of the rigid-duct cosine mode. The dimensionless quantities $E_{r}$, $E_{t}$ and $E_{abs}$ represent the fraction of the unit incident power that returns to the inlet, the fraction that emerges in the outlet, and the fraction lost inside the porous core and at the membranes. We emphasise that, because $E_{abs}$ is *defined* in Eq (27) as the power deficit, the closure


$$\begin{array}{c} E_{r}+E_{t}+E_{abs}=1 \end{array}$$ (28)

holds by construction and carries no validating power of its own; it is an accounting identity, not a check. The meaningful energy validation is instead performed by two independent computations, both reported in the results section.

First, the absorbed power is recomputed from a dissipation audit that never invokes Eq (27). The time-averaged axial power flux through a duct cross-section in an air-filled region is


$$\begin{array}{c} P(x):=\frac{\omega \rho }{2}\int_{-a}^{a}Im\{\phi^{*}(x,y)\;\partial_{x}\phi (x,y)\}\;dy, \end{array}$$ (29)

evaluated by numerical quadrature of the reconstructed fields, with the sign convention chosen so that the incident wave alone carries the positive flux $P_{inc}=\omega \rho ka$. Since the air-filled regions are lossless, *P*(*x*) is independent of *x* within each of $\Omega_{1}$, $\Omega_{2}$, $\Omega_{4}$ and $\Omega_{5}$, and the absorbed fraction obtained independently from the audit is


$$\begin{array}{c} E_{abs}^{flux}:=\frac{P_{2}-P_{4}}{P_{inc}}+\frac{P_{1}-P_{2}}{P_{inc}}+\frac{P_{4}-P_{5}}{P_{inc}}, \end{array}$$ (30)

where $P_{j}$ denotes the flux evaluated inside region $\Omega_{j}$. The first term is the power deposited in the porous core; the second and third terms are the net powers delivered to the left and right membranes, which must vanish separately because the membranes are conservative (real tension, no damping). Agreement of $E_{abs}^{flux}$ with $1-E_{r}-E_{t}$, and the separate vanishing of the two membrane terms, together constitute a non-circular energy validation of the solved amplitudes.

Secondly, the lossless limit provides a complementary check in which the conserved quantity is not defined as a deficit: when the porous propagation constant and the effective-density ratio are replaced by their real parts, every element of the structure is conservative and the identity $E_{r}+E_{t}=1$ – which is imposed nowhere in the linear systems – must emerge from the solved amplitudes alone.

The transmission loss


$$\begin{array}{c} TL:=-10\log_{10}(E_{t})\;\;(\text{dB}) \end{array}$$ (31)

recasts the transmitted fraction as a frequency-domain attenuation in decibels. Per-mode power fractions


$$\begin{array}{c} E_{r}^{\left(n\right)}:=\frac{|R_{n}{|}^{2}\;\eta_{n}\;X_{n}}{k\;X_{0}},\;E_{t}^{\left(n\right)}:=\frac{|T_{n}{|}^{2}\;\eta_{n}\;X_{n}}{k\;X_{0}}, \end{array}$$ (32)

quantify the contribution of each individual cut-on transverse mode to the global power partition and satisfy $E_{r}=\sum_{n}E_{r}^{\left(n\right)}$ and $E_{t}=\sum_{n}E_{t}^{\left(n\right)}$.

## 6 Numerical results and discussion

The numerical results below were computed for the parameters *a* = 0.05 m (duct half-width), *L* = 0.10 m (porous half-length), *c* = 343.5 m/s (ambient air sound speed), ρ=1.2043 kg/m^3^ (ambient air density), $\rho_{m}=0.1715$ kg/m^2^ (membrane surface mass density), *T* = 350.0 N/m (membrane tension, giving the membrane wave speed $c_{m}=45.18$ m/s), and the A-glass-fibre Delany–Bazley empirical constants tabulated in [29], namely


$$\begin{array}{c} \begin{array}{cccccccc} a_{1} & =0.2251, & a_{2} & =-0.5827, & a_{3} & =0.1443, & a_{4} & =-0.7088,\\ a_{5} & =0.0924, & a_{6} & =-0.7177, & a_{7} & =0.1457, & a_{8} & =-0.5951,\\ A_{1} & =1.857, & A_{2} & =1.687. \end{array} \end{array}$$

Following [29], the resistivity scaling is evaluated there with the ambient density in place of the bulk density of the fibrous skeleton, and the same convention is retained here for direct comparability with that cylindrical benchmark; it yields σ=2.541 N s m^–4^ and hence ξ=0.474f with *f* in Hz. The realised values of ξ therefore range from 23.7 at 50 Hz to 711 at 1.5 kHz in the main sweeps, and reach $5.7\times 10^{3}$ at the highest frequency (12 kHz) of the higher-mode studies. These values lie above the fitted window 0.01≤ξ≤1 of the original Delany–Bazley correlations [32]. In this regime the correlations of Eq (4) extrapolate smoothly toward a transparent medium, so the porous core behaves as a weakly dissipative equivalent fluid: at the reference frequency the realised constants are Γ=1.0030+0.0093i and τ=1.0049+0.0037i, and both tend to unity as the frequency increases. The porous core is therefore to be read throughout as a weakly resistive filler described by an empirical complex propagation constant and effective-density ratio of the Delany–Bazley form. The formulation and the solver accept arbitrary complex pairs of these constants, so a calibrated material characterisation – with the flow resistivity obtained from the bulk-density correlation of Kirby and Cummings [33], which returns ξ to the fitted window at audio frequencies – would change only the two complex constants supplied to the linear systems, leaving the method, the algorithm and the validation machinery untouched; the absolute absorption levels reported below are, however, tied to the parameter set stated above. The reference frequency used for the validation is $f_{ref}=500$ Hz with the truncation *N* = 16 transverse acoustic modes and *M* = 22 Galerkin modes.

Validation of the truncated solution proceeds in three steps: the point-wise matching conditions are reconstructed from the modal amplitudes, the truncation convergence is measured, and the energy audit of Eqs (29) and (30) is carried out. At the reference frequency the fluid normal velocity in the inlet at x=−2L is reconstructed from the modal coefficients of the membrane Galerkin system and overlaid against the membrane velocity $-i\omega \;U_{1}(y)$ in Fig 2(a); the two curves coincide to within line width over the entire interior of the cross-section. Fig 2(b) reconstructs the air-porous pressure continuity at the stationary interface x=−L, overlaying the real part of $\phi_{2}(-L,y)$ in the left air cavity against the real part of $\tau \;\phi_{3}(-L,y)$ in the porous core. The maximum relative residuals over the interior |*y*| < 0.9 *a* (which excludes the narrow Gibbs zone near the clamped membrane corners) are $5.26\times 10^{-4}$ for the membrane-velocity condition, $4.01\times 10^{-16}$ for the air-porous pressure condition, and $5.28\times 10^{-16}$ for the air-porous velocity condition; the last two are at machine precision because the air-porous matching is enforced in closed form through Eq (18), while the first decreases monotonically with the truncation.

**Fig 2:**
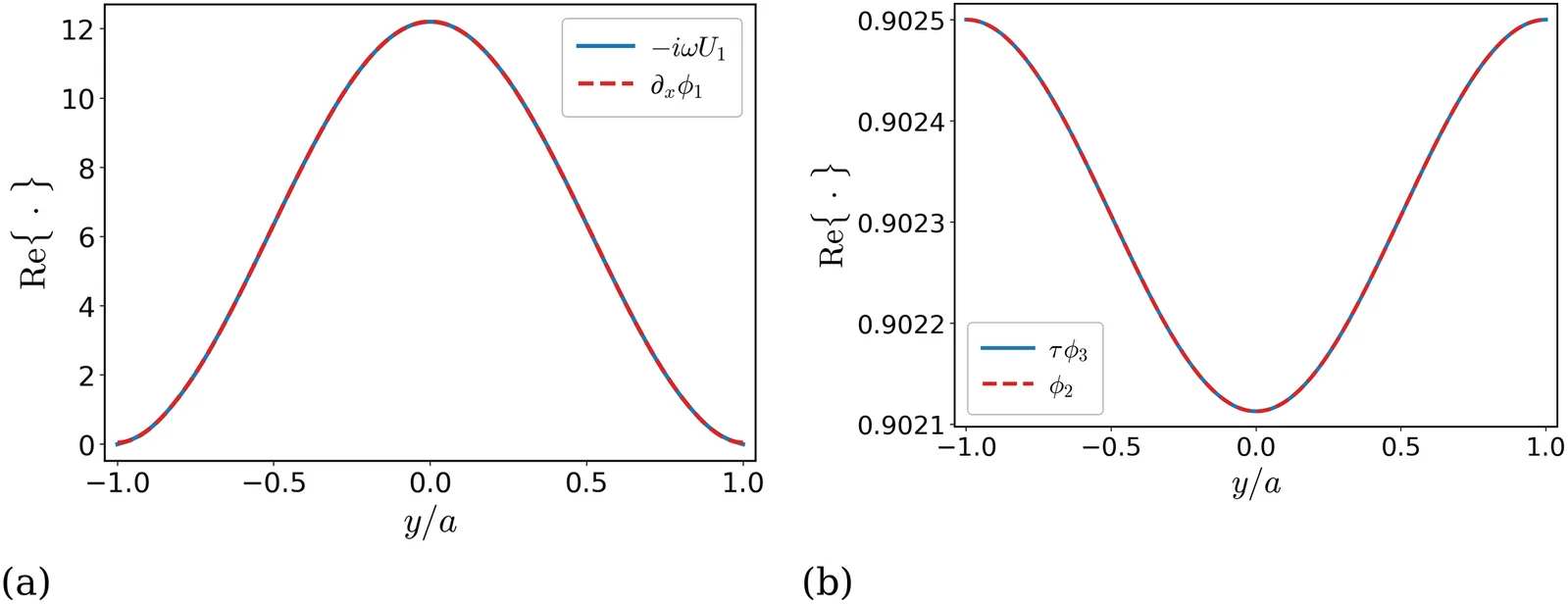
Pointwise validation of the matching conditions. Reference frequency $f_{ref}=500$ Hz with *N* = 16 acoustic transverse modes and *M* = 22 Galerkin modes; physical parameters *a* = 0.05 m, *L* = 0.10 m, *T* = 350.0 N/m. Panel (a): real part of the fluid normal velocity $\partial_{x}\phi_{1}(-2L,y)$ in the inlet (dashed) overlaid on the membrane velocity $-i\omega \;U_{1}(y)$ (solid). Panel (b): real part of $\phi_{2}(-L,y)$ in the left air cavity (dashed) overlaid on $\tau \;\phi_{3}(-L,y)$ in the porous core (solid). The closed-form elimination of Eq (18) enforces panel (b) to machine precision; panel (a) is convergent in the truncation. Physical interpretation: the membrane velocity matches the inlet fluid normal velocity – confirming the fluid-structure coupling of Eq (9) – and the air-porous pressure jump carries the correct factor τ from the Delany–Bazley filler.

Fig 3 reports the truncation convergence of the membrane-velocity residual at x=−2L: the error decreases monotonically from $4.54\times 10^{-3}$ at *N* = 6 to $9.06\times 10^{-5}$ at *N* = 40 (with the Galerkin truncation fixed at *M* = *N* + 8), with no sign of stagnation, so the truncation (*N*,*M*) = (16,22) used in all subsequent figures is conservative. The offset between the two truncations deserves a comment, since the convergence of coupled Galerkin–mode-matching schemes is known to depend on the ratio of the structural to the acoustic basis size. The sine basis of Eq (13) must resolve the trace of the truncated cosine expansion on the membrane, and the projection matrix of Eq (14) couples a sine mode of index *m* to cosine modes of every index of opposite parity, so a modest surplus of Galerkin modes over acoustic modes is the natural choice. The specific offset is uncritical here: at *N* = 16, varying the surplus M−N between 2 and 14 changes each of the three power fractions by less than $3\times 10^{-5}$ and leaves the membrane-velocity residual between $4.0\times 10^{-4}$ and $9.2\times 10^{-4}$, with no plateau or degradation; the values *M* = *N* + 8 (convergence study) and *M* = *N* + 6 (reference truncation) both sit comfortably inside this insensitive range.

**Fig 3:**
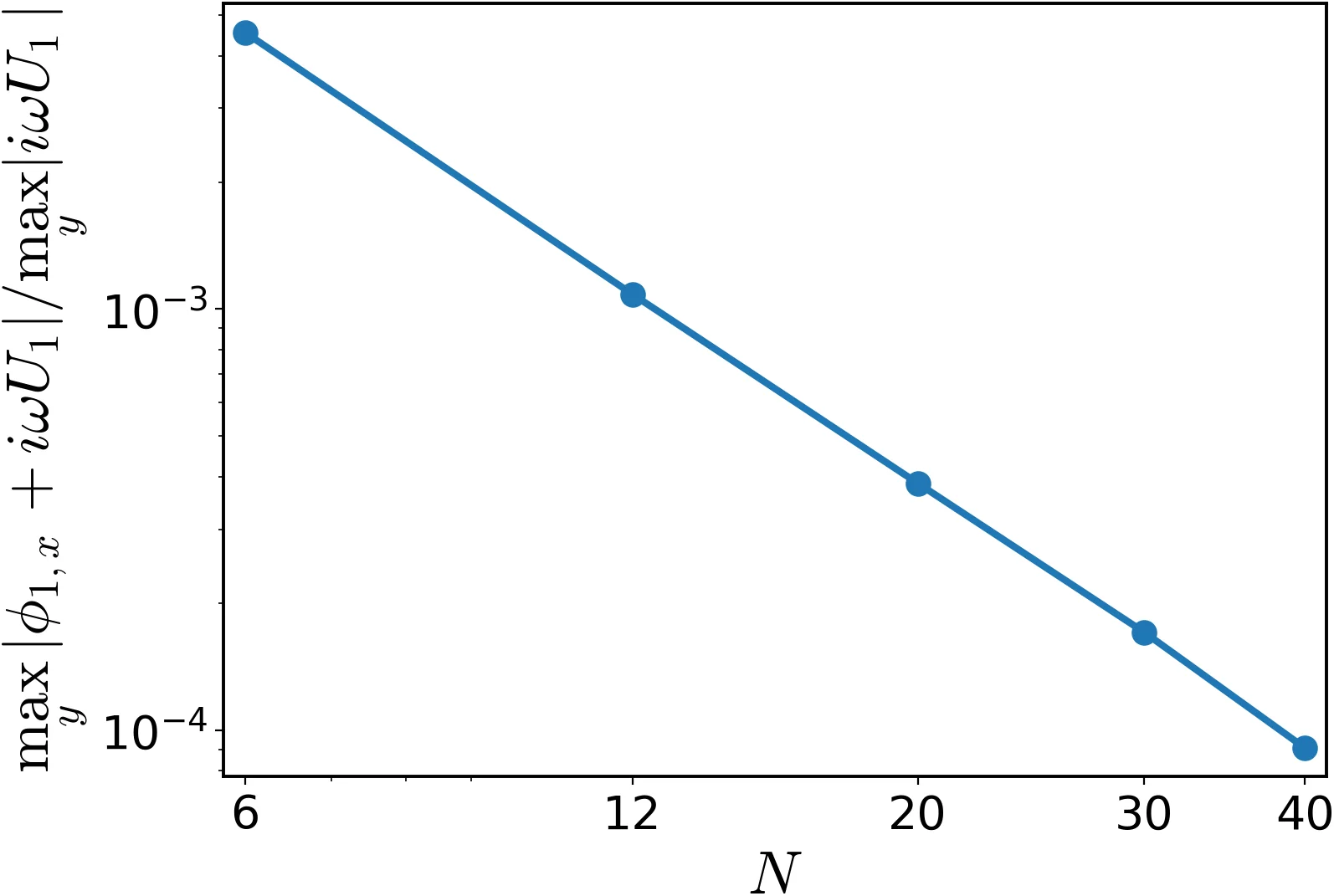
Truncation convergence of the membrane-velocity matching residual at x=−2L. The residual is plotted on a log–log scale against the acoustic transverse truncation *N*, with the Galerkin truncation fixed at *M* = *N* + 8 and *f* = 500 Hz, *a* = 0.05 m, *L* = 0.10 m, *T* = 350.0 N/m. The error decreases from $4.54\times 10^{-3}$ at *N* = 6 to $9.06\times 10^{-5}$ at *N* = 40, without stagnation. Physical interpretation: the truncated modal basis is genuinely convergent for the membrane-velocity matching; the algebraic decay rate is set by the corner singularity at the clamped membrane edges and is improved by Lanczos filtering in the field heatmaps.

The energy audit completes the validation. Because $E_{abs}$ in Eq (27) is defined as a deficit, the closure of Eq (28) is automatic and is not quoted as evidence. Instead, the flux audit of Eq (30) was evaluated by numerical quadrature (4000-point trapezoidal rule in the transverse coordinate, central finite differences in the axial coordinate) at stations inside the inlet, the two air cavities and the outlet. At the reference frequency the independently computed absorbed fraction agrees with $1-E_{r}-E_{t}$ to within $1.1\times 10^{-11}$, and the same agreement (to within 10^–11^) holds at every frequency spot-checked across the band (196–1500 Hz, including the absorption peak at 1224 Hz); the net power delivered to each membrane is below $1.1\times 10^{-11}$ of the incident power, confirming that the membranes are conservative and that the entire deficit is deposited in the porous core. Complementarily, in the lossless limit – the porous propagation constant and effective-density ratio replaced by their real parts – the identity $E_{r}+E_{t}=1$, which is imposed nowhere in the linear systems, emerges from the solved amplitudes to within $6.7\times 10^{-16}$ at all fourteen frequencies of a uniform sweep of 100–1400 Hz. Together these two computations provide the non-circular counterpart of Eq (28).

With the solver validated, attention turns to the redistribution of propagating power between higher-order transverse modes. The first higher-order cut-on for the rigid duct in the inlet and outlet is $f_{1}=c/(4a)\approx 1.72$ kHz; the second cut-on is at $f_{2}=c/(2a)\approx 3.44$ kHz. Per-mode reflected and transmitted power fractions $E_{r}^{\left(n\right)}=|R_{n}{|}^{2}\eta_{n}X_{n}/(kX_{0})$ and $E_{t}^{\left(n\right)}=|T_{n}{|}^{2}\eta_{n}X_{n}/(kX_{0})$ satisfy $E_{r}=\sum_{n}E_{r}^{\left(n\right)}$ and $E_{t}=\sum_{n}E_{t}^{\left(n\right)}$ over cut-on modes. For a plane-wave incidence ($n_{inc}=0$) on the *y*-symmetric structure the projection coefficients $R_{mn}$ in Eq (14) vanish whenever *m* + *n* is even; only odd-*m* Galerkin modes are excited, and the reflected/transmitted modal amplitudes $R_{n},T_{n}$ are therefore identically zero for *odd n* regardless of frequency. Consequently the first higher mode that can carry energy is *n* = 2, which activates above *f*_2_. Fig 4 reports the modal decomposition over the broadband sweep f∈[60,3500] Hz: below *f*_1_ all the energy is carried by the *n* = 0 mode; between *f*_1_ and *f*_2_ mode *n* = 1 is propagating but carries zero power by the parity selection rule above; above *f*_2_ mode *n* = 2 activates and begins to carry a small share of the partition near the top of the swept range.

**Fig 4:**
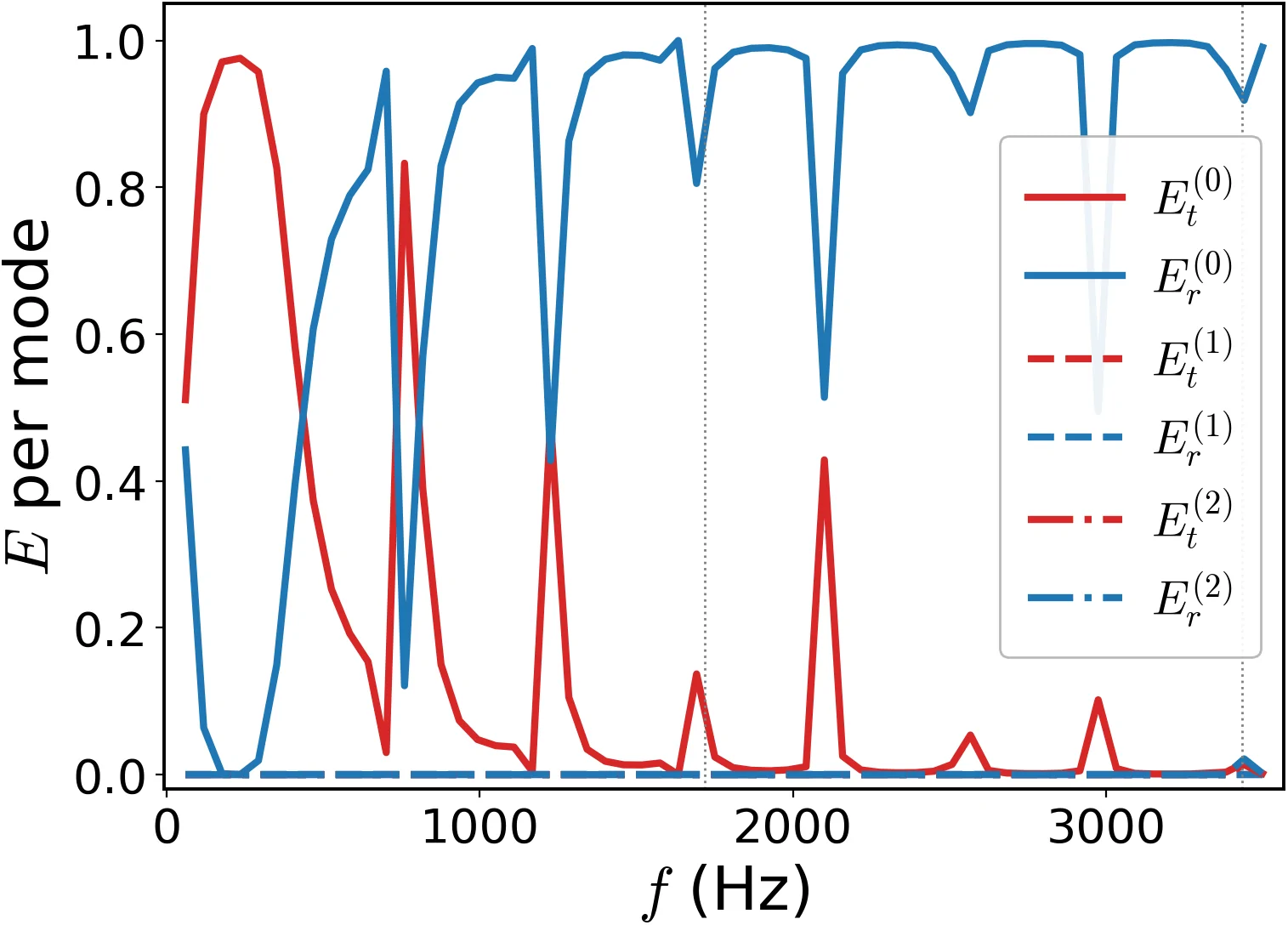
Per-mode power decomposition across the broadband sweep. Per-mode reflected $E_{r}^{\left(n\right)}$ and transmitted $E_{t}^{\left(n\right)}$ power fractions for *n* = 0 (solid), *n* = 1 (dashed) and *n* = 2 (dotted), across the broadband sweep f∈[60,3500] Hz at *a* = 0.05 m, *L* = 0.10 m, *T* = 350.0 N/m, *N* = 24 transverse modes, *M* = 32 Galerkin modes. The vertical grey dotted lines mark the cut-on frequencies *f*_1_ = *c*/(4*a*) and *f*_2_ = *c*/(2*a*). Physical interpretation: below *f*_1_ only the plane-wave channel carries energy. The *n* = 1 curves stay identically zero across the whole sweep because the parity selection rule of Eq (14) ($R_{mn}\neq 0$ only when *m* + *n* is odd) prevents the *y*-symmetric structure from coupling the plane-wave incidence into odd transverse modes. The first higher mode that activates is *n* = 2, above *f*_2_, marking the onset of multi-mode propagation.

To visualise the modal filtering directly the structure is driven by a unit-amplitude wave incident in a single higher-order rigid-duct mode $n_{inc}\in \{2,3,4\}$ above that mode’s cut-on $f_{n}:=nc/(4a)$; the incident-mode flux normalisation in Eq (27) becomes $\eta_{n_{inc}}X_{n_{inc}}$. Fig 5 reports the steady-state field magnitude |ϕ(x,y)| over the full 14*L* window in *x* for $n_{inc}=2$ at the reference $f_{H}=8000$ Hz (above the cut-on $f_{4}\approx 6870$ Hz of every mode up to *n* = 4), computed with *N* = 32 acoustic transverse modes and *M* = 40 Galerkin modes; the modal sums are dressed with the Lanczos sigma factors $\sigma_{n}=sinc(n/N)$ for visual Gibbs suppression. Figs 6 and 7 then give a four-panel diagnostic composite for the higher incident modes $n_{inc}=3$ and $n_{inc}=4$, reporting the membrane displacement profile at a reference frequency together with the three power fractions $E_{abs}$, $E_{r}$, $E_{t}$ on a frequency sweep above the corresponding cut-on (the truncations (*N*,*M*) = (24,32) and (28,36) are used respectively to resolve the higher-mode Galerkin response). In all figures solid blue vertical lines mark the membrane planes x=±2L and red dashed lines mark the air-porous interfaces x=±L where applicable.

**Fig 5:**
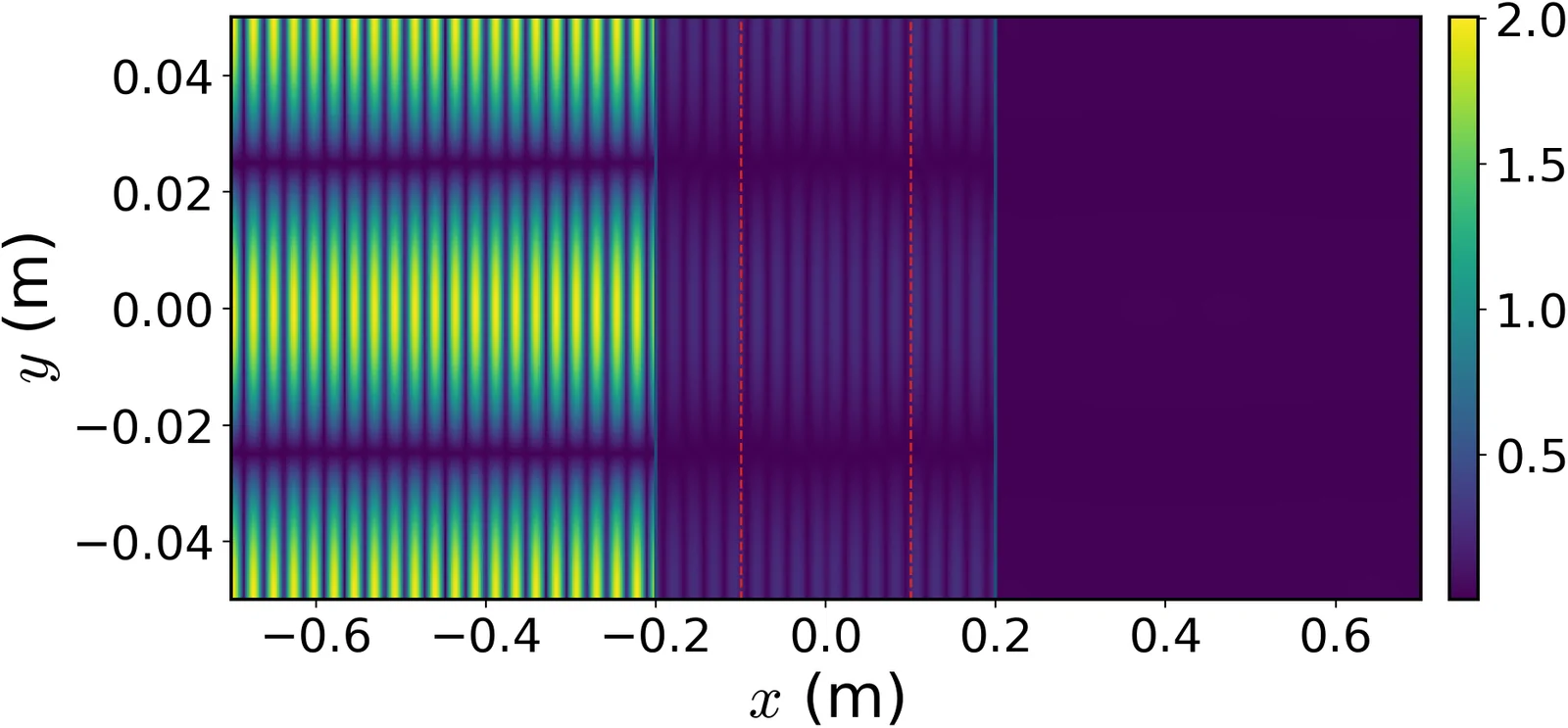
Steady-state field magnitude for a higher-order incident mode. Steady-state |ϕ(x,y)| for a unit-amplitude incident wave in rigid mode $n_{inc}=2$ at $f_{H}=8000$ Hz, computed with *N* = 32, *M* = 40 and Lanczos-filtered for display. Physical parameters *a* = 0.05 m, *L* = 0.10 m, *T* = 350.0 N/m. The inlet shows the standing-wave interference between the incident mode-2 wave (two transverse lobes) and its reflection from the left membrane; the porous core attenuates the field; the outlet carries only a residual amplitude. Physical interpretation: the membrane and porous core together act as a strong low-pass spatial filter on the higher modes, with the reflection coefficient $E_{r}$ already exceeding 0.99 at $n_{inc}=2$.

**Fig 6:**
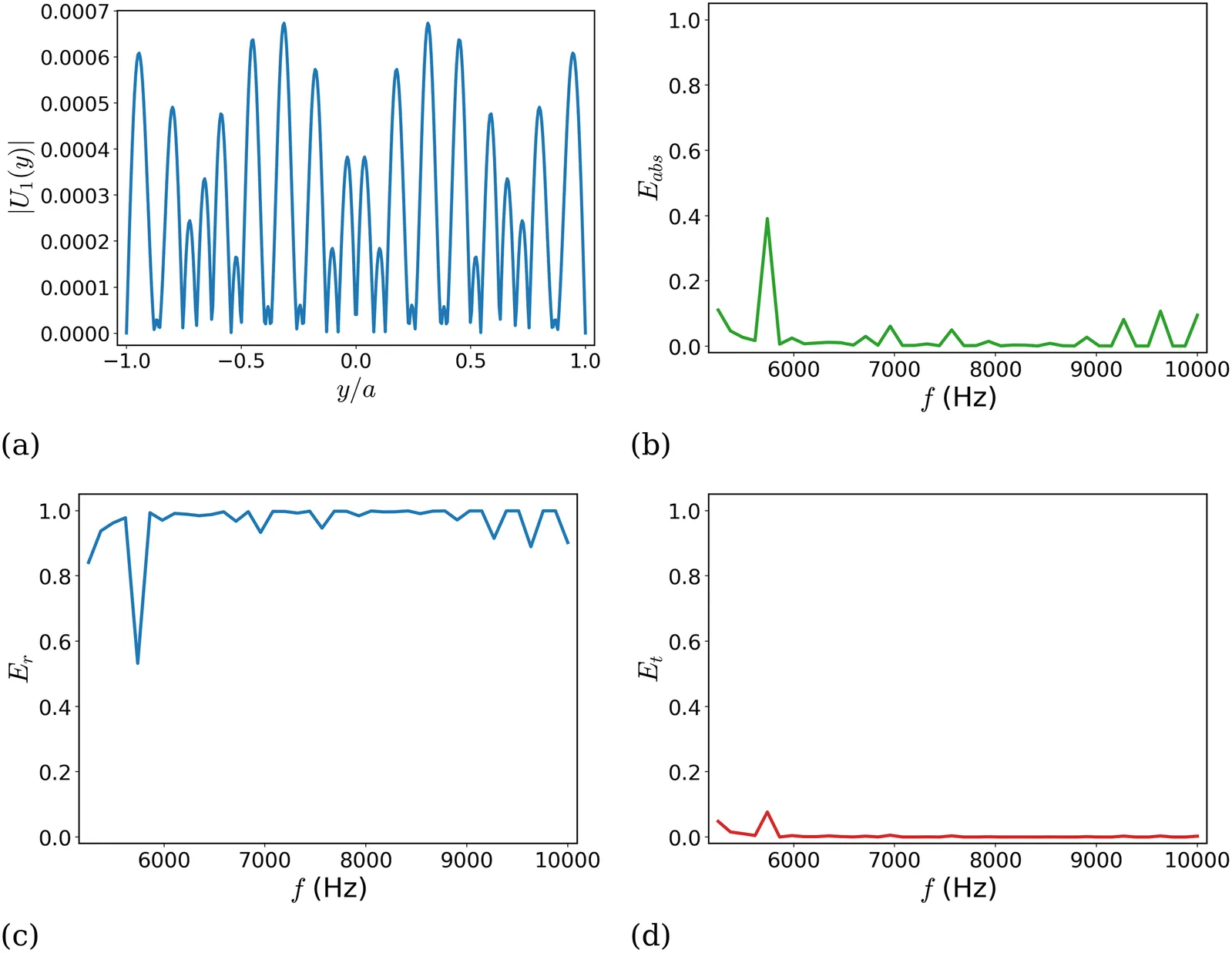
Higher-mode response for an incident wave in rigid mode $n_{inc}=3$. Physical parameters *a* = 0.05 m, *L* = 0.10 m, *T* = 350.0 N/m; truncation *N* = 24, *M* = 32. Panel (a): magnitude |*U*_1_(*y*)| of the physical membrane displacement at $f_{ref,3}=6500$ Hz. Panel (b): absorbed-power fraction $E_{abs}(f)$ across the sweep $f\in [f_{3}+100,10000]$ Hz with $f_{3}=3c/(4a)\approx 5152$ Hz. Panel (c): reflected-power fraction $E_{r}(f)$ on the same sweep. Panel (d): transmitted-power fraction $E_{t}(f)$ on the same sweep.

**Fig 7:**
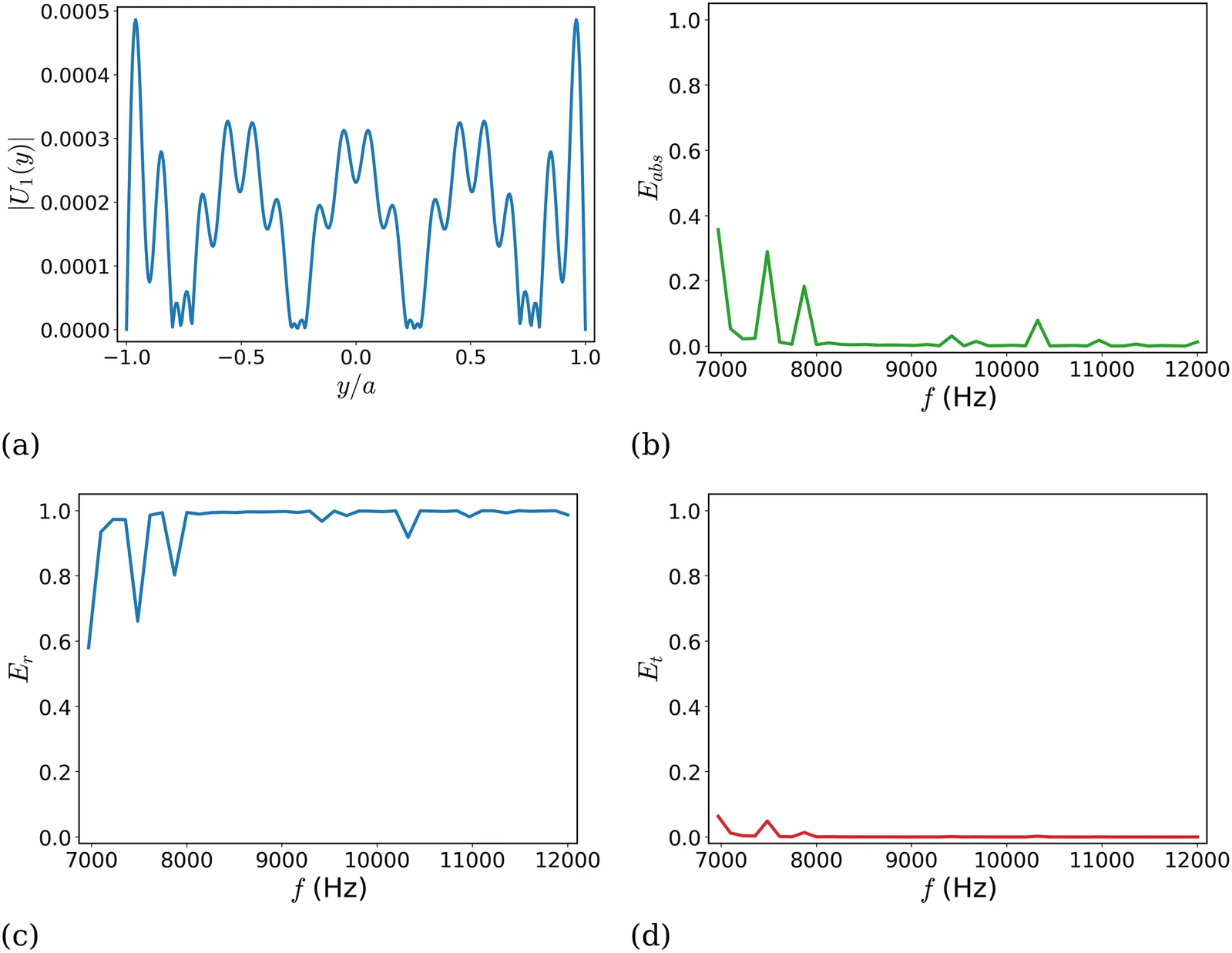
Higher-mode response for an incident wave in rigid mode $n_{inc}=4$. Physical parameters *a* = 0.05 m, *L* = 0.10 m, *T* = 350.0 N/m; truncation *N* = 28, *M* = 36. Panel (a): magnitude |*U*_1_(*y*)| of the physical membrane displacement at $f_{ref,4}=8500$ Hz. Panel (b): $E_{abs}(f)$ across $f\in [f_{4}+100,12000]$ Hz with $f_{4}=c/a\approx 6870$ Hz. Panel (c): $E_{r}(f)$. Panel (d): $E_{t}(f)$. Across Figs 5–7 every higher-order incident mode is strongly reflected – the reflected fraction exceeds 0.96 throughout each sweep and rises above 0.99 well beyond the corresponding cut-on – the modal-filtering signature of the device.

Beyond the single steady-state snapshot of Fig 5, we characterise the higher-mode response by four diagnostic quantities: the physical membrane displacement profile $U_{1}(y):=\frac{1}{2}[U^{s}(y)+U^{a}(y)]$ (m), where $U^{s}(y)=\sum_{m}w_{m}^{s}\;\psi_{m}(y)$ and $U^{a}(y)$ is its anti-symmetric counterpart, and the three unit-normalised power fractions $E_{abs}$, $E_{r}$, $E_{t}$ of Eq (27) swept across frequency. Fig 6 reports the four panels for the incident mode $n_{inc}=3$ at the carrier reference $f_{ref,3}=6500$ Hz (above the cut-on $f_{3}=3c/(4a)\approx 5152$ Hz). Panel (a) shows the membrane vibration profile excited by the mode-3 pressure jump – the highly oscillatory shape reflects the strong coupling between the higher-order incident wave and the Galerkin modes of the membrane. Panel (b) reports $E_{abs}(f)$ on the swept range, exhibiting a narrow absorption peak near the lowest membrane-cavity resonance accessible to mode $n_{inc}=3$ and otherwise staying small. Panel (c) reports $E_{r}(f)$ – the structure reflects the bulk of the higher-mode incident energy. Panel (d) reports $E_{t}(f)$ – the transmitted fraction is minute across most of the sweep (median below 10^–3^) and rises only to a narrow peak near 0.08 at the lowest membrane-cavity resonance, confirming that the membrane-porous filter strongly rejects the higher-order incident mode.

Fig 7 reports the analogous four-panel response for the incident mode $n_{inc}=4$ at the higher reference $f_{ref,4}=8500$ Hz (above the cut-on $f_{4}=c/a\approx 6870$ Hz). The membrane displacement profile in panel (a) is even more oscillatory than the mode-3 case, the absorbed band in panel (b) shifts to higher frequency, and the reflected and transmitted fractions in panels (c) and (d) confirm that the higher-order incident mode is again almost entirely reflected, with the transmitted fraction peaking near 0.06 at the membrane-cavity resonance.

The parametric dependence of the absorbed fraction is exposed by sweeping three independent design parameters in turn – the porous half-length *L*, the membrane tension *T*, and the duct half-width *a* – with the remaining parameters held at their reference values. Each sweep is reported as a frequency-versus-parameter heatmap of the absorbed fraction $E_{abs}$ accompanied by three representative frequency slices. Fig 8 shows that the absorbed fraction remains negligible at low frequencies for every *L* considered (below a few hundred hertz, where the porous core is acoustically thin), and then organises into curved bands of enhanced $E_{abs}$ at higher frequencies. The bands correspond to the longitudinal resonances of the porous segment, $Re(s_{0})\cdot 2L\approx n\pi$; their frequencies decrease as 1/*L*, which is exactly the curvature visible in the heatmap. The propagation loss inside the core scales as $\exp(-2\;Im(s_{0})\;L)$, so the envelope of the absorption peaks grows almost linearly with *L*. The frequency slices in Fig 8(b) confirm both effects: at *L* = 0.05 m no clear resonance appears below 1500 Hz; at *L* = 0.10 m a prominent absorption peak emerges near 1200 Hz; at *L* = 0.18 m a sequence of absorption peaks is established with amplitudes growing as *L* increases.

**Fig 8:**
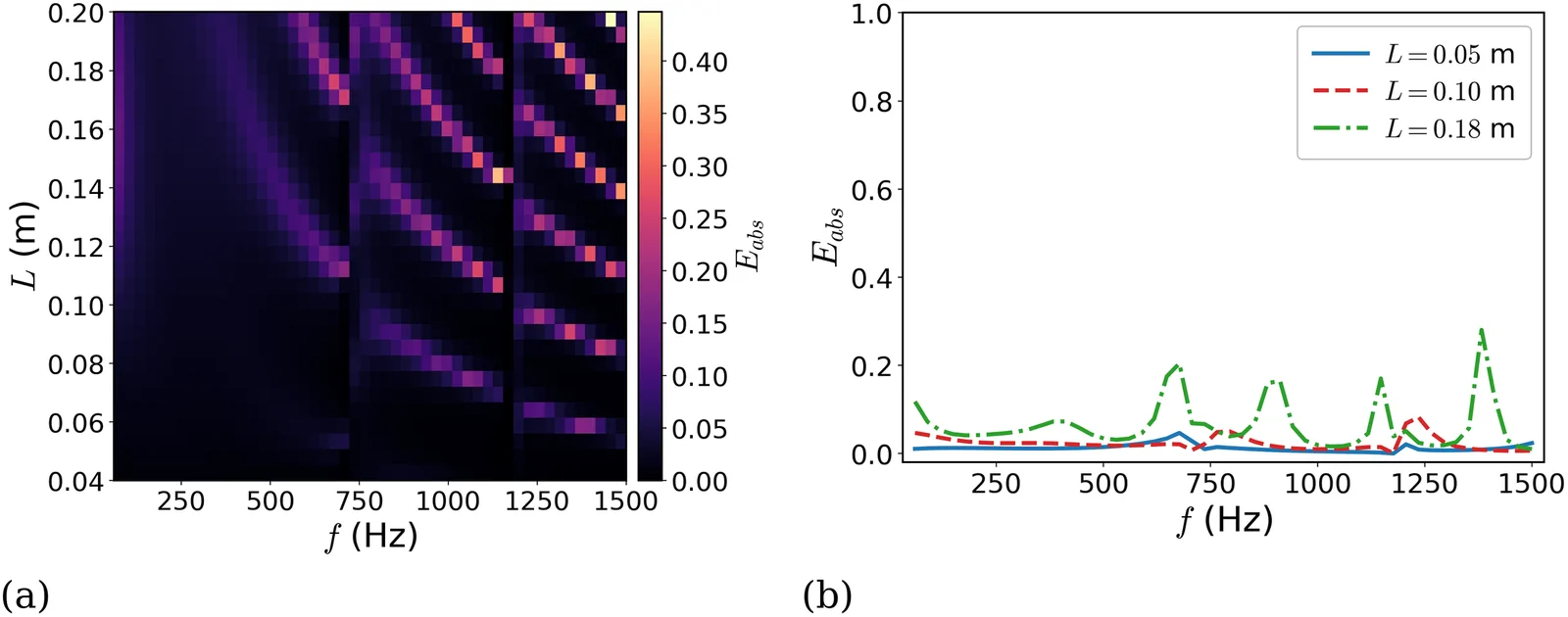
Influence of the porous half-length on the absorbed fraction. Parameters *a* = 0.05 m, *T* = 350.0 N/m, frequency sweep f∈[60,1500] Hz, length sweep L∈[0.04,0.20] m, *N* = 12, *M* = 18. Panel (a): heatmap of $E_{abs}$ over the (*f*, *L*) plane. Panel (b): frequency slices at *L* = 0.05, 0.10, 0.18 m. Physical interpretation: the curved bands in (a) are longitudinal cavity resonances of the porous segment shifting as 1/*L*; the absorption envelope grows with *L* because the in-core propagation loss scales as $\exp(-2\;Im(s_{0})\;L)$.

Fig 9 reports the analogous tension sweep. Two features appear simultaneously: nearly vertical bright bands near f≈760 and 1200 Hz are acoustic cavity resonances of the membrane-cavity-porous-cavity-membrane ensemble whose spectral locations depend only weakly on *T*; a diagonal stripe climbing monotonically with *T* is the in-plane structural resonance of the membrane, $f_{mem}\propto \sqrt{T/\rho_{m}}$, modified by fluid loading. When the structural branch crosses an acoustic branch the two resonances hybridise and the absorbed fraction reaches its parametric maximum at the intersection point. The frequency slices in Fig 9(b) show that the absorbed level is broadband-flat at the intermediate tension *T* = 350 N/m, while the low (*T* = 100 N/m) and high (*T* = 700 N/m) tensions yield similar absorption profiles with peaks near the acoustic cavity resonances; the non-monotonic behaviour with *T* is the signature of the membrane-cavity hybridisation rather than a monotonic stiffness effect.

**Fig 9:**
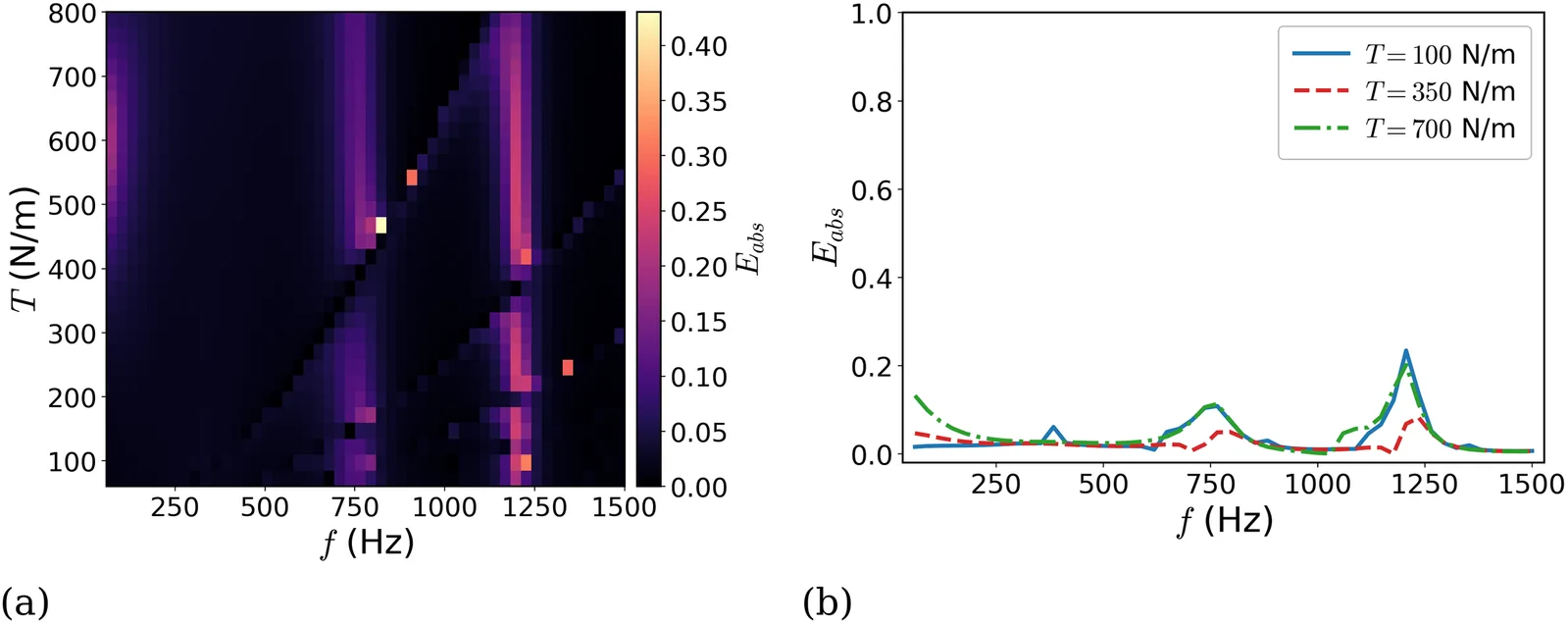
Influence of the membrane tension on the absorbed fraction. Parameters *a* = 0.05 m, *L* = 0.10 m, f∈[60,1500] Hz, T∈[60,800] N/m, *N* = 12, *M* = 18. Panel (a): heatmap of $E_{abs}$ over the (*f*, *T*) plane. Panel (b): frequency slices at *T* = 100, 350, 700 N/m. Physical interpretation: the diagonal feature in (a) is the structural membrane resonance branch $f_{mem}\propto \sqrt{T/\rho_{m}}$; its intersections with the nearly vertical acoustic-cavity branches define the parametric absorption optima where the reactive (membrane) and resonant (cavity) attenuations hybridise.

Fig 10 reports the absorbed fraction as the duct half-width *a* varies from 0.025 to 0.10 m. The vertical bright bands again correspond to acoustic cavity resonances; their slight curvature with *a* reflects the dependence of the transverse cosine eigenvalues $\alpha_{n}=n\pi /(2a)$ on the duct width, which in turn modulates the axial wavenumbers $\eta_{n}=\sqrt{k^{2}-\alpha_{n}^{2}}$ and shifts the cut-on frequencies $f_{n,cut}=nc/(4a)$. At smaller *a* the mode-1 cut-on lies higher in the audio band, leaving the plane-wave regime intact across a wider frequency span; at larger *a* higher-order acoustic modes participate in the matching and produce additional spectral structure. The frequency slices in Fig 10(b) confirm that the wider duct (*a* = 0.08 m) exhibits an additional absorption peak between 800 and 1100 Hz that is absent in the narrower duct (*a* = 0.035 m), consistent with the per-mode decomposition of Fig 4.

**Fig 10:**
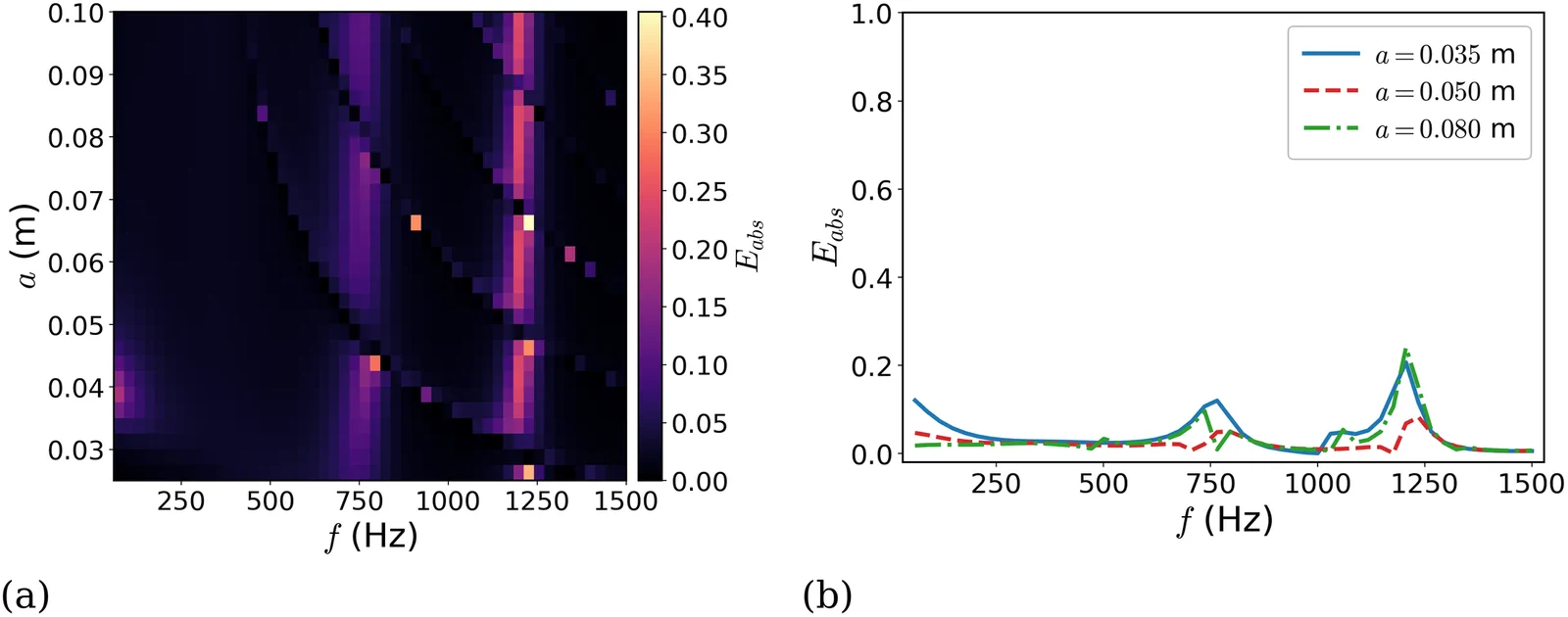
Influence of the duct half-width on the absorbed fraction. Parameters *L* = 0.10 m, *T* = 350.0 N/m, f∈[60,1500] Hz, a∈[0.025,0.10] m, *N* = 12, *M* = 18. Panel (a): heatmap of $E_{abs}$ over the (*f*, *a*) plane. Panel (b): frequency slices at *a* = 0.035, 0.05, 0.08 m. Physical interpretation: the slight curvature of the vertical bands reflects the dependence of the transverse-mode cut-on frequencies on *a*; the wider duct admits an additional propagating mode in the audio band and adds spectral structure correspondingly.

Fig 11 shows the magnitude of the steady-state velocity potential at *f* = 500 Hz over a wide 14*L* window in *x* that spans all five regions. The reconstructed field is dressed with Lanczos sigma factors $\sigma_{n}=sinc(n/N)$ during the visualisation so that the modal-truncation Gibbs ringing at the clamped membrane corners is suppressed. The incoming wave establishes a clear interference pattern in the inlet between the incident and reflected components; the porous core attenuates the field by approximately a factor of two in amplitude; and the outlet carries a nearly plane transmitted wave of reduced amplitude. Fig 12 gives the corresponding view on a narrower 6*L* window centred on the structure, in which the air-porous impedance jump τ at x=±L is visible as a faint amplitude discontinuity.

**Fig 11:**
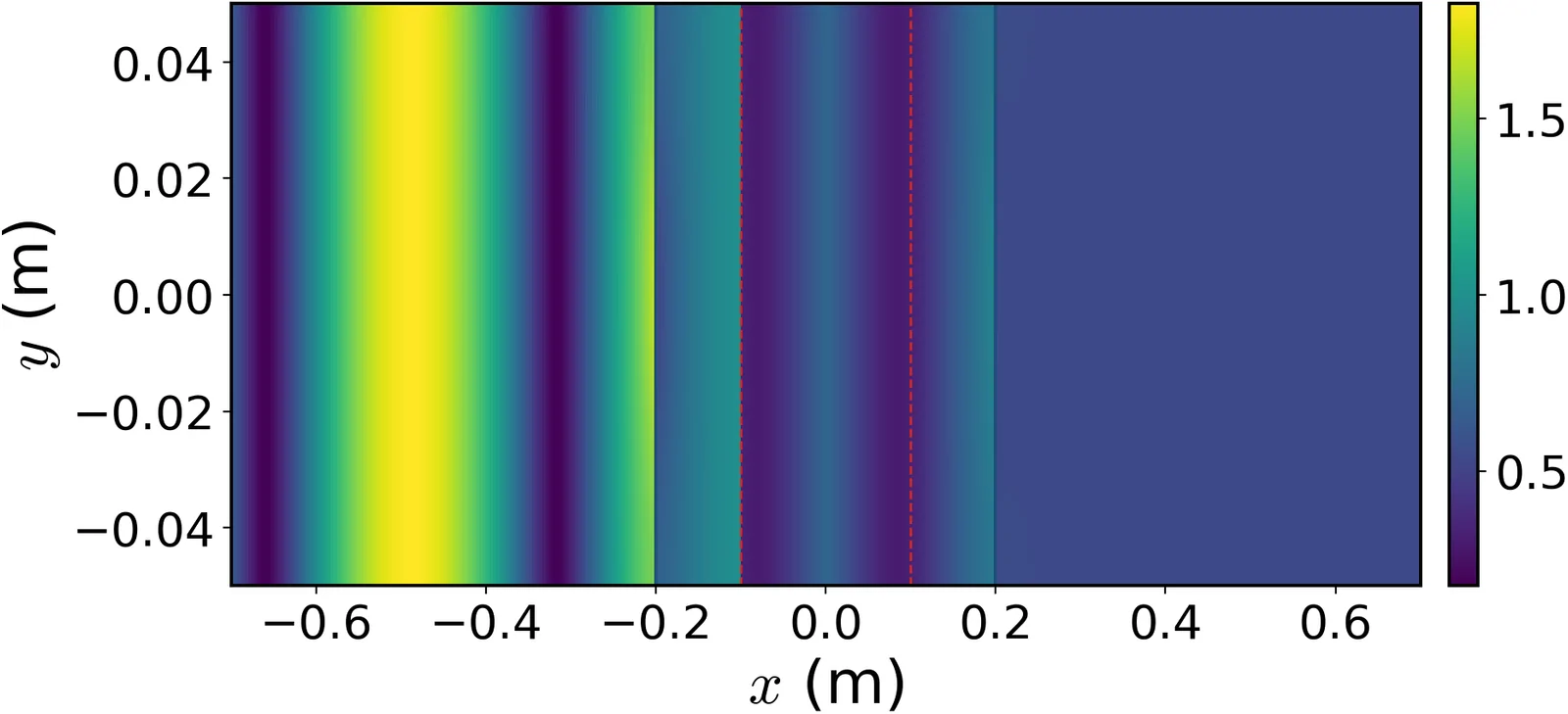
Steady-state field over the full geometry. Magnitude |ϕ| of the steady-state velocity potential at *f* = 500 Hz over the full 14*L* window in *x*, with all five regions $\Omega_{1}$–$\Omega_{5}$ visible; *a* = 0.05 m, *L* = 0.10 m, *T* = 350.0 N/m, *N* = 24, *M* = 32. The modal series is Lanczos-filtered with $\sigma_{n}=sinc(n/N)$ for visual Gibbs suppression at the clamped corners. Solid blue vertical lines mark the membrane planes x=±2L; red dashed lines mark the air-porous interfaces x=±L. Physical interpretation: the inlet carries an interference pattern between incident and reflected waves; the porous core attenuates the field by roughly a factor of two in amplitude; the outlet carries a nearly plane transmitted wave.

**Fig 12:**
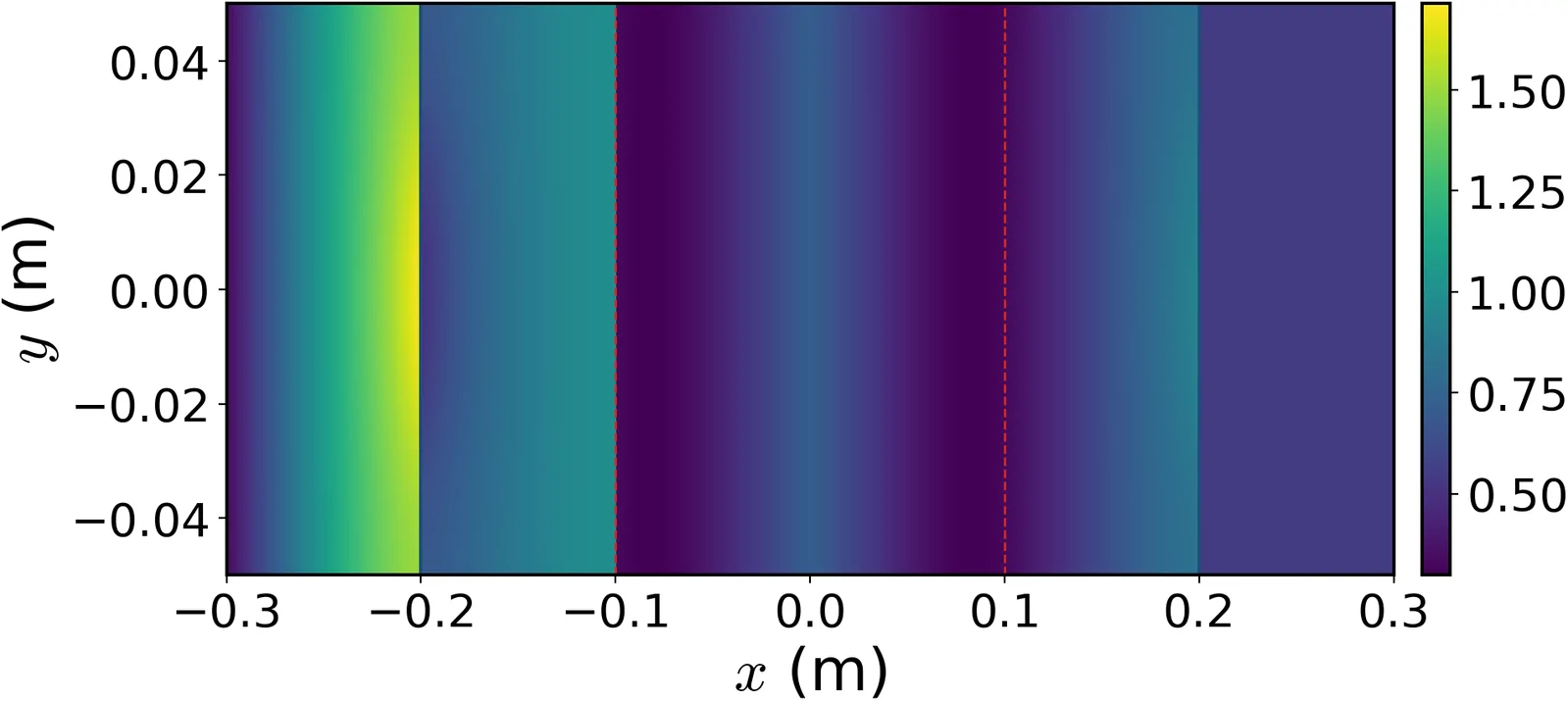
Steady-state field near the structure. Magnitude |ϕ| of the steady-state velocity potential at *f* = 500 Hz over a narrower 6*L* window centred on the structure; *a* = 0.05 m, *L* = 0.10 m, *T* = 350.0 N/m, *N* = 24, *M* = 32 with Lanczos filtering. Physical interpretation: the amplitude jumps at x=±L that are barely visible in Fig 11 are now resolved as a step in the magnitude controlled by the porous impedance factor τ of Eq (6).

Fig 13 reports six snapshots of the time-dependent total field obtained from Eq (26) on the same 14*L* window, with the same Lanczos filtering applied during each per-frequency solve in the wavepacket reconstruction. The incident Gaussian wavepacket is centred at the reference wavenumber $k_{0}=k(500\;Hz)=9.15$ rad/m with bandwidth $\sigma_{k}=1.0$ rad/m on the $N_{k}=40$ wavenumber grid stated earlier; as established there, the envelope centre of the incident packet crosses the left membrane plane at *t* = 0, which makes the quoted snapshot times self-contained. In panel (a) the leading edge of the pulse is still propagating in the inlet. In panel (b) *t*he pulse has reached the left membrane and a small reflected wavefront has appeared in the inlet. By panel (c) – the instant at which the envelope centre sits on the left membrane plane – the leading half of the pulse already occupies the porous core and is visibly attenuated. In panel (d) it has emerged from the right membrane with substantially reduced amplitude. Panels (e) and (f) follow the long-time ring-down: a reflected pulse retreats upstream, a transmitted pulse propagates downstream, and a slowly decaying resonant field trapped between the two membranes loses energy through repeated interaction with the porous core.

**Fig 13:**
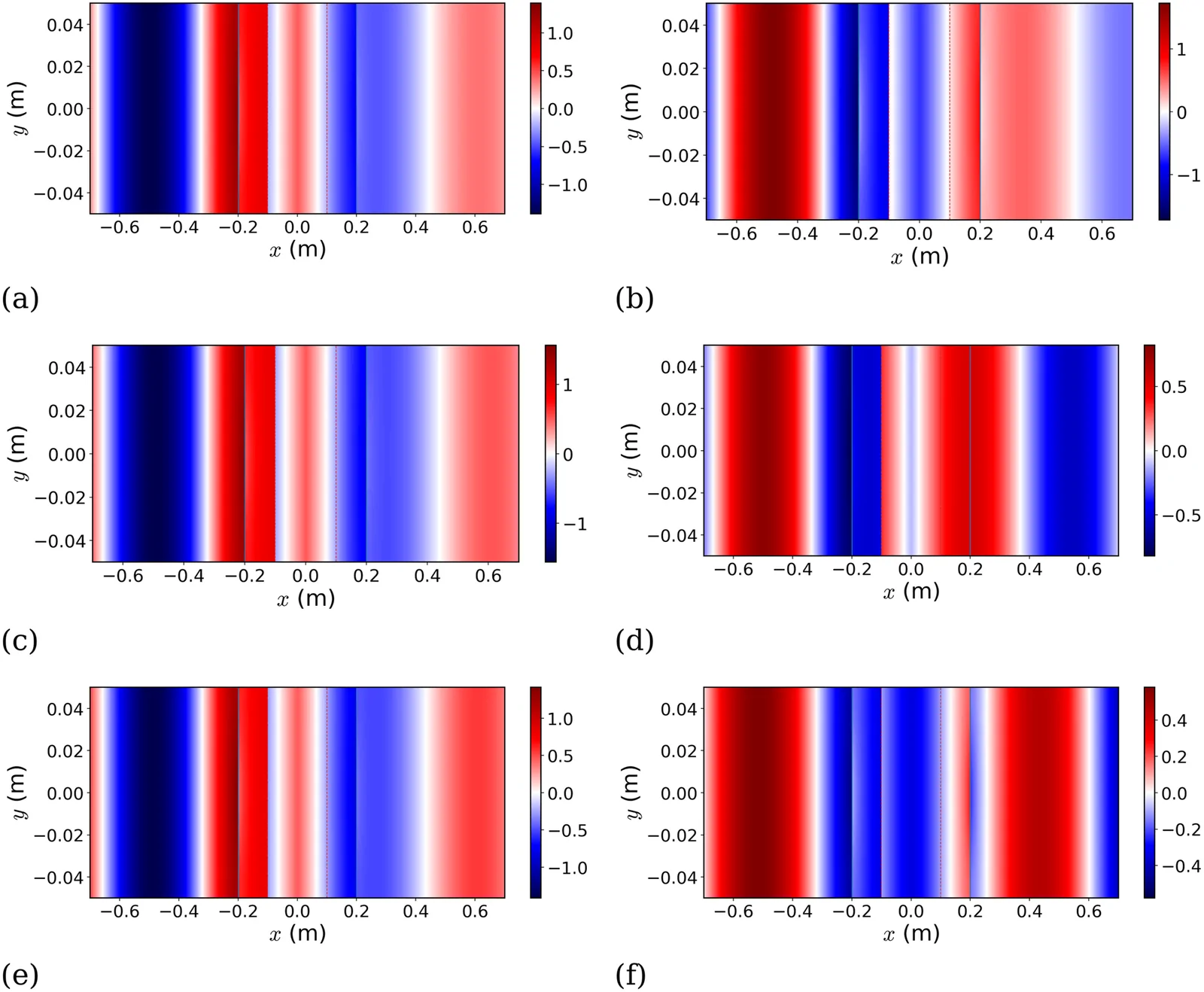
Time-domain reconstruction of the total velocity potential. Gaussian wavepacket excitation centred at *f*_0_ = 500 Hz (*k*_0_ = 9.15 rad/m, bandwidth $\sigma_{k}=1.0$ rad/m, $N_{k}=40$ wavenumber points) on the full 14*L* window; *a* = 0.05 m, *L* = 0.10 m, *T* = 350.0 N/m, *N* = 16, *M* = 22, Lanczos-filtered per-frequency solves. The envelope centre of the incident packet crosses the left membrane plane x=−2L at *t* = 0. Snapshot times: (a) t=−2.0 ms, (b) t=−0.8 ms, (c) *t* = 0, (d) *t* = +0.8 ms, (e) *t* = +2.0 ms, (f) *t* = +3.5 ms. Solid blue vertical lines mark the membrane positions; red dashed lines mark the air-porous interfaces. Physical in*t*erpretation: a reflected pulse retreats upstream, a transmitted pulse propagates downs*t*ream, and a slow ring-down *t*rapped be*t*ween *t*he membranes decays through repeated interaction with the porous core.

To quantify the energy stored in (and absorbed by) the porous filler during the transit, we define the time-dependent absorbed-power proxy


$$\begin{array}{c} W_{a}(t):=\int_{-L}^{L}\;\;\int_{-a}^{a}\varphi^{2}(x,y,t)\;dx\;dy\;\;({\text{m}}^{6}/{\text{s}}^{2}), \end{array}$$ (33)

where φ(x,y,t) is the real-valued time-domain velocity potential obtained from Eq (26) with the Gaussian wavepacket envelope of Eq (25); since φ is real, no modulus is needed in the integrand. The integral runs over the porous core |*x*| < *L*, |*y*| < *a*. With the potential in m^2^/s, the squared integrand carries m^4^/s^2^ and the double integral over the core area yields the stated unit m^6^/s^2^. We stress that $W_{a}(t)$ is a *field-intensity proxy* for the energy content of the core, not the acoustic energy density itself – the latter would combine $|\nabla \varphi {|}^{2}$ with $(\partial_{t}\varphi {)}^{2}/c^{2}$ – and it is likewise distinct from the dimensionless steady-state absorbed fraction $E_{abs}$ of Eq (27). For the narrow-band wavepackets used here the two energy-like measures are monotonically related, so $W_{a}(t)$ serves as a faithful comparative indicator of the instantaneous field energy stored in (and being dissipated by) the porous filler: it peaks while the pulse is inside the core and decays as the residual energy is radiated out through the membranes. The transient-energy studies below use the bandwidth $\sigma_{k}=2.5$ rad/m on the $N_{k}=80$ grid stated earlier, yielding a temporal pulse width $\sigma_{t}\approx 1/(c\;\sigma_{k})\approx 1.2$ ms that is short compared with the round-trip travel time through the porous core.

Fig 14 reports $W_{a}(t)$ for three carrier frequencies $f_{0}\in \{300,500,900\}$ Hz of the incident Gaussian wavepacket while the other parameters are held at the reference values. The low-frequency carrier *f*_0_ = 300 Hz produces a single broad transient with a smooth envelope (the structure is in its transmission-dominated band, so the pulse enters the core, deposits energy, and exits without strong re-reflection); the reference *f*_0_ = 500 Hz is intermediate and modest in amplitude; the higher carrier *f*_0_ = 900 Hz produces multiple closely-spaced peaks of comparable height to the 300 Hz envelope because the structure has entered its reflection-dominated regime, so the pulse interacts repeatedly with the two membranes and traverses the core several times before the residual energy is evacuated by dissipation and radiation. The peak amplitudes of the 300 Hz and 900 Hz traces are comparable; the difference between the two regimes lies in the temporal shape – a broad single lobe at the low carrier versus a multi-peak ring-down at the high carrier.

**Fig 14:**
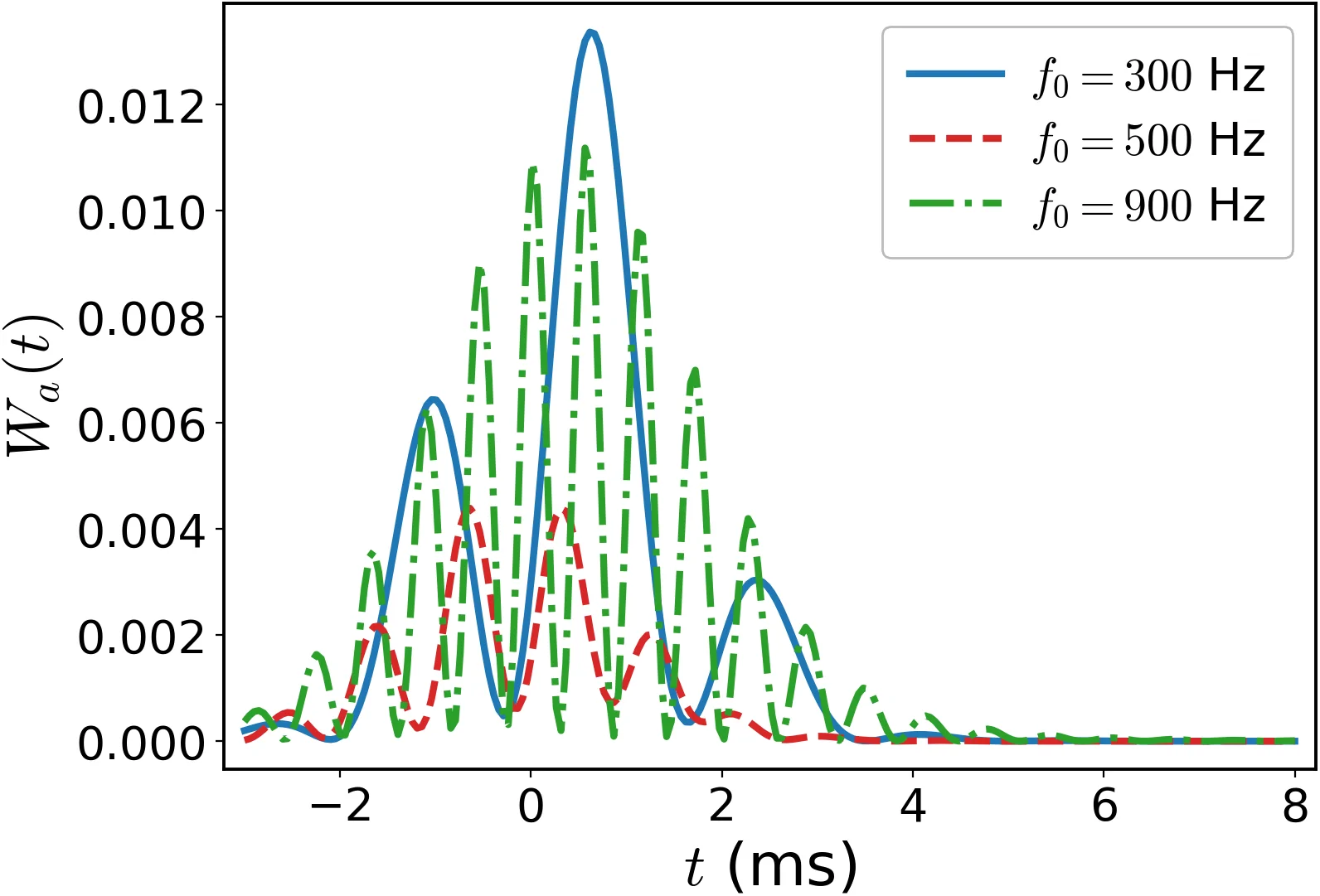
Transient core energy for three carrier frequencies. Time-variation of the absorbed-power proxy $W_{a}(t)$ of Eq (33) for three carrier frequencies of the incident Gaussian wavepacket: *f*_0_ = 300 Hz (blue), *f*_0_ = 500 Hz (red), *f*_0_ = 900 Hz (green). Physical parameters *a* = 0.05 m, *L* = 0.10 m, *T* = 350.0 N/m; truncation *N* = 16, *M* = 22; pulse bandwidth $\sigma_{k}=2.5$ rad/m. Physical interpretation: the low carrier produces a single broad transient; the high carrier produces multiple closely-spaced peaks of comparable height, corresponding to repeated reflections of the pulse from the two membranes before the residual energy is dissipated and radiated.

Fig 15 reports $W_{a}(t)$ for three porous half-lengths L∈{0.05,0.10,0.18} m at the fixed carrier *f*_0_ = 500 Hz. The longest porous segment (*L* = 0.18 m) traps a substantially larger fraction of the incident pulse energy and produces a ringing signature with several well-resolved peaks spanning 0–3 ms; the two shorter lengths produce only modest transients. The mechanism is the linear scaling of the single-trip propagation loss $\exp(-2\;Im(s_{0})\;L)$ in the porous core: a longer core retains the pulse over more round trips before the combined action of the dissipative spectrum and the membrane reactance evacuates the stored energy.

**Fig 15:**
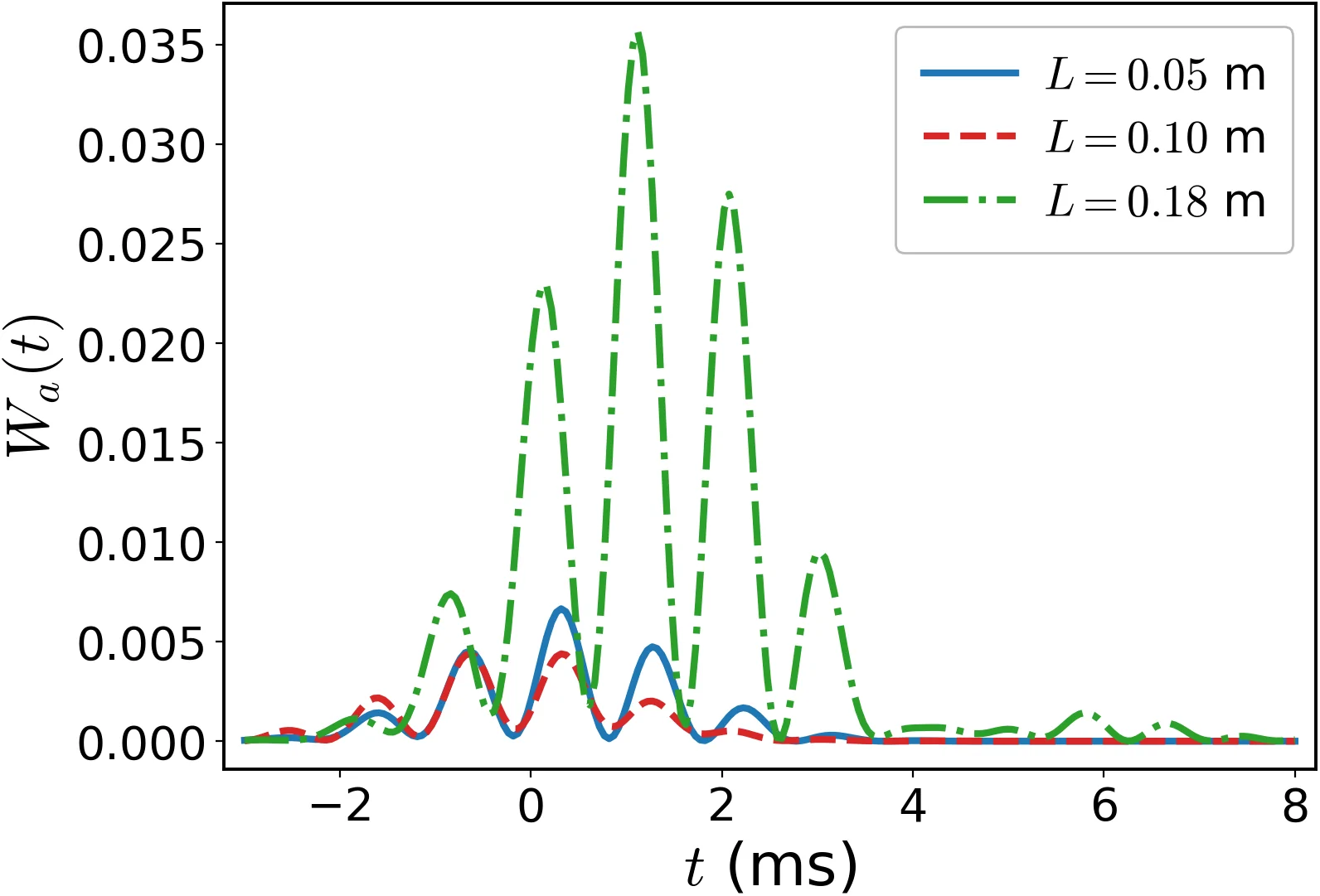
Transient core energy for three porous half-lengths. Time-variation of $W_{a}(t)$ for three porous half-lengths L∈{0.05,0.10,0.18} m at the reference carrier *f*_0_ = 500 Hz. Other physical parameters *a* = 0.05 m, *T* = 350.0 N/m; truncation *N* = 16, *M* = 22; pulse bandwidth $\sigma_{k}=2.5$ rad/m. Physical interpretation: the longer porous core stores substantially more incident-pulse energy and exhibits ringing across several millisecond-scale peaks before the residual energy is dissipated.

Fig 16 reports $W_{a}(t)$ for three membrane tensions T∈{100,350,700} N/m at the fixed carrier *f*_0_ = 500 Hz and the nominal porous half-length. The intermediate tension *T* = 350 N/m yields the smallest core transient, consistent with the steady-state observation that this tension lies in a broadband-flat absorbed region (Fig 9). The low and high tensions both produce larger transients with discernible ring-down structure, signalling that the membrane-cavity hybridisation is closer to a resonance for those tensions.

**Fig 16:**
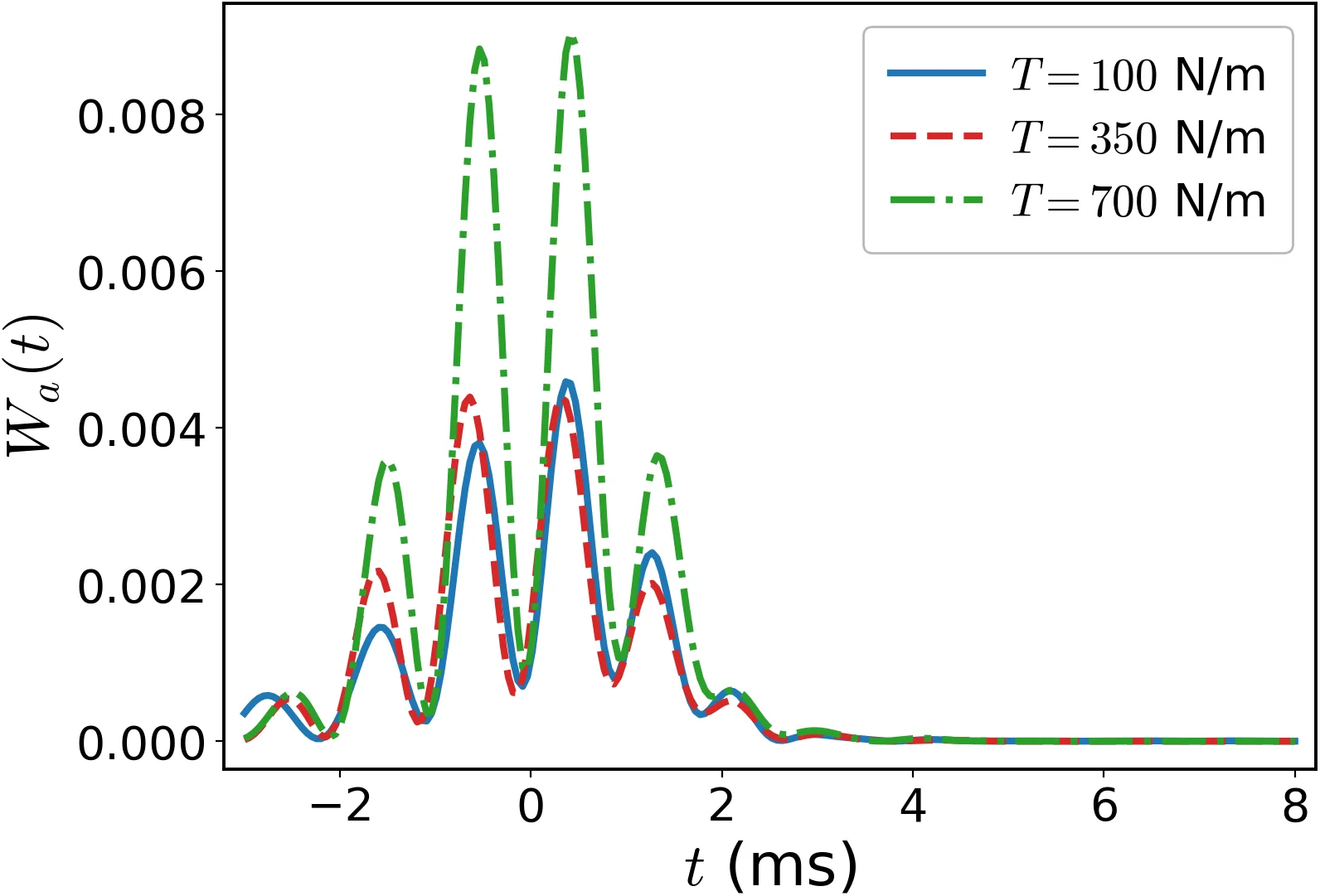
Transient core energy for three membrane tensions. Time-variation of $W_{a}(t)$ for three membrane tensions T∈{100,350,700} N/m at the reference carrier *f*_0_ = 500 Hz. Other physical parameters *a* = 0.05 m, *L* = 0.10 m; truncation *N* = 16, *M* = 22; pulse bandwidth $\sigma_{k}=2.5$ rad/m. Physical interpretation: the intermediate tension *T* = 350 N/m yields the smallest core transient (broadband-flat absorbed band of Fig 9); the low and high tensions both produce larger transients with longer ring-downs, signalling proximity to a membrane-cavity hybrid resonance.

## 7 Conclusion

A complete time-dependent semi-analytical treatment has been formulated, solved, validated, and reported for an incident acoustic pulse propagating through a rectangular waveguide whose central region contains a dissipative porous core flanked by two clamped flexible membrane strips. The formulation combines a decomposition into symmetric and anti-symmetric subproblems, a Galerkin treatment of the membrane discontinuity, and a Delany–Bazley model of the porous filler, developed directly for the Cartesian rectangular setting. The Gaussian-wavepacket Fourier reconstruction of the time-dependent field has been organised as a single matrix-vector product per snapshot through the time-independent spatial matrix, so each new time instant incurs only one matrix–vector product once the family of frequency-domain solutions has been precomputed. Validation gates the whole pipeline: a point-wise interface reconstruction, a truncation convergence study, and an independent energy audit – in which the absorbed power recomputed from a dissipation integral over the porous core agrees with the power deficit of the solved amplitudes, the net power delivered to each conservative membrane vanishes, and the lossless limit returns the incident power in full – must all succeed before any quantitative result is emitted. Frequency-domain results document a low-frequency transmission band, an intermediate mixed regime, and a high-frequency reflection-dominated regime, with a localised absorption peak that identifies the membrane-cavity co-tuning. Time-domain snapshots visualise how the wavepacket loses energy to the membranes and to the porous core as it crosses the structure, showing reflection, attenuated transmission and post-transit ring-down in a single sequence of frames. The combined geometry-and-time treatment is, to the best of our knowledge, new and provides both a quantitative design tool and a qualitative insight into how broadband transients interact with hybrid reactive–dissipative filters.

## Supporting information

S1 Appendix From the membrane equation to the Galerkin systems. Intermediate algebra leading from the membrane boundary value problem of Eq (8) to the Galerkin systems of Eqs (20) and (21): the Galerkin projection of the membrane equation, the two velocity conditions projected on the cosine basis, the closed-form elimination of the porous amplitudes, the assembled trace jump at the membrane, the corresponding chain for the anti-symmetric subproblem, and the orientation convention that makes the right-membrane problem the exact mirror image of the left one.
(TEX)
