# Supplementary material for: Computational time-dependent acoustic scattering in a rectangular membrane–porous waveguide
Source: PLoS One. 2026 Sep 30;21(9):e0358579. doi: 10.1371/journal.pone.0358579 (PMC13626333; doi:10.1371/journal.pone.0358579)
Supplement: Supplementary file 1 [file pone.0358579.s005.pdf]

Response to the Reviewers

Manuscript: Computational time-dependent acoustic scattering in a rectangular membrane–porous waveguide
PLOS ONE

We are thankful to the reviewers and the handling editor for their valuable time and constructive comments, which have led to a substantially strengthened revision. Below, each reviewer comment is reproduced in full, followed by our response. In the marked-up copy of the revised manuscript, all new or modified text is highlighted in blue; deleted text is simply removed rather than shown, so the marked-up copy reads as the final text with the changes visible at a glance. In accordance with both reviewers' explicit statements, no figure has been added, removed or altered; all changes are confined to the text, the analysis quoted in the text, and the bibliography. The manuscript has been prepared in the PLOS ONE LaTeX template (clean copy with captions only, per the journal instructions; the marked-up copy embeds the unchanged figures for reading convenience), and the reference list has been renumbered in order of first appearance – because virtually every entry changed number, the list appears without highlighting in the marked-up copy. In addition, the one unpublished preprint cited in the original submission has been replaced by a published, peer-reviewed article, so that every entry in the reference list is now a citable published source.

Since new equations were added, equation numbers below refer to the *revised* manuscript, with old numbers in brackets where helpful; the most useful correspondences are old (4) → new (4), old (8) → new (8), old (18)–(19) → new (20)–(22), old (21) → new (24), old (23) → new (26), old (24)–(25) → new (27)–(28), and old (28) → new (33).

Reviewer 1

General comments. *The manuscript combines a mode-matching/Galerkin formulation with a wavepacket–Fourier time reconstruction for a membrane–porous rectangular filter. The topic is relevant, the formulation is largely sound and the presentation is generally clear, but the central validation claim is logically circular as written, key material data are missing for reproducibility, and the novelty relative to closely related work needs sharpening. The figures are appropriate as they stand and no changes to them are requested; my comments concern the text and the analysis. I therefore recommend major revision.*

Response. We thank the reviewer for this accurate summary and for three criticisms – circularity of the energy check, missing material data, and novelty – that we accept in full. Each is addressed below; in accordance with the reviewer's statement, no figure has been altered.

Comment 1. *In Eq. (24) the absorbed fraction is defined as* $E_{abs} := 1 - E_r - E_t$*, so identity (25) holds by construction; reporting* $|E_r + E_t + E_{abs} - 1|$ *below* 10^{-15} *in Sec. 6 therefore validates nothing. Please compute the absorbed power independently (dissipation integral over the porous core plus membrane input power) and quote the resulting residual in the text, or at least verify* $E_r + E_t = 1$ *in the lossless limit (real* Γ, τ*).*

Response. The reviewer is entirely right: as written, the closure claim was circular, and it has been removed as evidence. We have implemented *both* remedies the reviewer proposes, and the revised manuscript quotes each with its residual.

(i) *Independent dissipation audit.* The time-averaged axial power flux carried across a duct section is now defined in the manuscript (new Eq (29)) and is evaluated by direct numerical quadrature

of the reconstructed fields – a 4000-point trapezoidal rule across the section combined with central finite differences along the axis – at stations inside the inlet, the two air cavities and the outlet. This computation never invokes the modal power identity. The flux drop across the porous core, together with the power fed into the two membranes (new Eq (30)), reproduces the absorbed fraction obtained from the solved amplitudes to within 1.1×10^{-11} of the incident power at the reference frequency of 500 Hz, and to within 10^{-11} at every frequency spot-checked across the band (196, 508, 1004, 1224 and 1500 Hz, including the absorption peak). The net power delivered to each membrane is separately below 1.1×10^{-11} of the incident power, as it must be for conservative (real-tension, undamped) membranes, so the entire deficit is deposited in the porous core.

(ii) *Lossless limit.* With the porous propagation constant and the effective-density ratio replaced by their real parts, the reflected and transmitted fractions – on which no conservation statement is imposed anywhere in the linear systems – are found to sum to the incident power to within 6.7×10^{-16} at all fourteen frequencies of a uniform sweep of 100–1400 Hz.

The section formerly titled "Conserved power identity" is now "Power identities and independent energy audit"; it states explicitly that the closure of Eq (28) [old (25)] "holds by construction and carries no validating power of its own; it is an accounting identity, not a check". The abstract, introduction, results and conclusion have been updated so that the energy validation is everywhere described as the independent audit plus the lossless-limit check. The verification script that produces these numbers (`revision_checks.py`) has been added to the reproducibility archive of the paper.

Comment 2. *In Eq. (4), $\sigma = A_1 \rho^{A_2}$ is presented as the flow resistivity with ρ declared as the air density; the standard empirical relation uses the bulk density of the fibrous material, a different quantity. Please correct the symbol, and report the numerical values of A_1, A_2, a_1–a_8 and the resulting σ actually used.*

Response. The reviewer is correct, and we have corrected the presentation and disclosed every number. The formulation section now states that for fibrous media the flow resistivity is conventionally estimated from the *bulk density* of the fibrous skeleton – not from the air density – through the standard power-law scaling, citing the experimental characterisations collected by Kirby and Cummings (new ref. [33], *Appl. Acoust.* 1999;56(2):101–125); the Delany–Bazley correlations themselves are now cited to the original source (new ref. [32]). The results section reports the complete numerical parameter set actually used: all eight curve-fit coefficients of the Delany–Bazley correlations and both constants of the resistivity scaling are listed explicitly in a dedicated display. It also states plainly that, following the cylindrical benchmark study (ref. [29]) with which we maintain direct comparability, the scaling was evaluated there with the ambient air density in place of the bulk density, which yields the resistivity value actually used, 2.541 $\mathrm{N\,s\,m^{-4}}$; the dimensionless Delany–Bazley frequency parameter then equals 0.474 times the frequency in Hz. The physical implications are addressed under the next comment.

Comment 3. *The frequency sweeps extend to 10–12 kHz for the higher-mode studies. Please state the range of $\xi = f\rho/\sigma$ realized and confirm it stays within the accepted validity window of the Delany–Bazley correlations.*

Response. The realized range is now stated explicitly: the Delany–Bazley parameter runs from 23.7 at 50 Hz to 711 at 1.5 kHz in the main sweeps, and reaches 5.7×10^3 at 12 kHz in the higher-mode studies. We must be candid rather than confirmatory: these values lie *above* the fitted window of the original correlations (0.01 to 1), and the revised manuscript says so directly. In this regime the correlations extrapolate smoothly toward a transparent medium, so the porous core behaves as

a weakly dissipative equivalent fluid; at the reference frequency both realized material constants lie within one percent of their transparent-medium values and tend to unity as the frequency grows. The revised text therefore instructs the reader to interpret the core throughout as a weakly resistive filler described by an empirical complex propagation constant and effective-density ratio of Delany–Bazley form, and notes that the formulation and the solver accept an arbitrary pair of complex material constants: a calibrated material characterisation, with the resistivity obtained from the bulk-density correlation of Kirby and Cummings – which returns the parameter to the fitted window at audio frequencies – would change only the two complex constants supplied to the linear systems, leaving the method, the algorithm and the validation machinery untouched. The absolute absorption levels are stated to be tied to the disclosed parameter set. We believe this transparent statement, together with the full disclosure under Comment 2, is the scientifically honest resolution, and we thank the reviewer for pressing on it.

Comment 4. *The passage from Eq. (8) to the Galerkin systems (18)–(19) is very compressed; a short appendix with the intermediate algebra, including the orientation convention of the pressure jump at the right membrane, is needed so signs and coefficients can be verified.*

Response. Done, in full. A new appendix ("From the membrane equation to the Galerkin systems", new Eqs (34)–(41)) records every intermediate step: the Galerkin projection of the membrane equation, the two velocity conditions projected on the cosine basis, the closed-form elimination of the porous amplitudes, the assembled trace jump at the membrane, and the same chain for the anti-symmetric subproblem. In carrying this out we also made the anti-symmetric system fully explicit in the main text (new Eq (21)): the previous wording – which asserted an analogous linear system with the coefficient families merely relabelled – was imprecise, because the bounded combination of those families changes form between the symmetric and the anti-symmetric case; the revised text and the appendix now state the exact form. Regarding the orientation of the pressure jump at the right membrane, the formulation section now writes out the right-membrane boundary value problem explicitly (new Eqs (10)–(11)) instead of asserting an unstated analogy: each membrane's displacement is measured positive *toward the chamber interior*, whereupon the driving jump is the interior-side minus the exterior-side pressure on both faces, with the matching signs in the fluid coupling on either membrane. The appendix proves that with this convention the right-membrane problem is the exact mirror image of the left one, notes the equivalent alternative convention (displacement measured along the duct axis, with the forcing reversed accordingly), and records that in the mirror decomposition the right-membrane conditions are satisfied identically once the left-membrane conditions hold in both parity subproblems – which the point-wise reconstruction verifies a posteriori.

Comment 5. *Since Ref. [24] already applies the same wavepacket–Fourier reconstruction to a rectangular chamber, the introduction should state more precisely what is methodologically new here beyond replacing resistive sheets by membranes plus a porous core (e.g., the clamped-sine Galerkin coupling and the closed-form R_{mn}).*

Response. Agreed. The introduction now contains a dedicated paragraph ("It is worth stating precisely what separates the present study. . . ") drawing the methodological line exactly where the reviewer indicates: in both of the closest antecedents – the rigid corner scatterers of Aldosri and Meylan (ref. [30]) and the resistive wire-mesh perforates of Lawrie and Afzal (ref. [31]) – the interface conditions are algebraic relations between the acoustic traces, so each frequency solve is mode matching alone with no structural unknowns; here each frequency solve is a coupled fluid–structure problem in which the membrane displacement is an additional unknown field with its own differential equation and clamped edges. The paragraph then names the specific new ingredients:

the clamped-sine Galerkin basis satisfying both edges identically, the closed-form projection matrix of the cosine modes onto that basis, the exact two-term elimination that absorbs the porous-core impedance so that the solved system has only the dimension of the membrane basis, and the parity selection rule governing which transverse modes a symmetric filter can pass. It is written without mathematical symbols, in keeping with the symbol-free introduction.

Reviewer 2

General comments. *The formulation is consistent, the pointwise matching reconstruction and the truncation convergence study are convincing, and the parametric and time-domain results are clearly interpreted. The figures adequately support the text and no changes or additions to them are requested. A number of small corrections and clarifications listed below, all confined to the text, should be addressed before acceptance.*

Response. We thank the reviewer for the positive assessment and for ten precise, constructive corrections; all ten are implemented, and no figure has been altered.

Comment 1. *The units of $W_a(t)$ in Eq. (28) are stated as m^4/s^2, but with φ in m^2/s the double integral yields m^6/s^2; the stated unit is that of the integrand only. Please correct.*

Response. Correct – the stated unit was that of the integrand. New Eq (33) now carries $\mathrm{m}^6/\mathrm{s}^2$, and the following sentence spells out the bookkeeping: with the potential in m^2/s the squared integrand carries $\mathrm{m}^4/\mathrm{s}^2$, and the double integral over the core area contributes the remaining m^2.

Comment 2. *Since $\varphi(x, y, t)$ in Eq. (28) is real-valued, the modulus signs are redundant; also clarify in the text that $|\varphi|^2$ is a field-intensity proxy rather than the acoustic energy density, or use a density based on $|\nabla\varphi|^2$ and $(\partial_t\varphi)^2/c^2$.*

Response. Both done, following the reviewer's first option. The integrand of new Eq (33) is now written as the plain square of the real-valued time-domain field, with an explicit remark that no modulus is needed. The text then states that the quantity is "a field-intensity proxy for the energy content of the core, not the acoustic energy density itself" – the latter would combine the squared field gradient with the squared time derivative of the field – notes that it is likewise distinct from the steady-state absorbed fraction, and adds that for the narrow-band wavepackets used here the two energy-like measures are monotonically related, which is what justifies using the proxy for the comparative transit studies.

Comment 3. *The final sentence of the abstract ends with "an algebraic conservation identity satisfied", which is grammatically incomplete; please rephrase.*

Response. Rephrased – and, in view of Reviewer 1's first comment, the sentence now describes the strengthened validation rather than the circular identity. It reads: "The block linear system is validated by point-wise reconstruction of pressure and velocity continuity at every matching plane, by a truncation convergence study, and by two independent energy checks: the absorbed power fraction is recomputed from a dissipation audit of the power fluxes entering and leaving the porous core, and the lossless limit of the porous model is shown to return the incident power in full."

Comment 4. *Reference [24] is an unpublished preprint cited without year or identifier; complete the bibliographic data or explicitly mark it as unpublished.*

Response. We have gone further than marking it. The unpublished preprint has been removed from the manuscript altogether, so that every entry in the reference list is now a published, citable source. In its place we cite the peer-reviewed treatment of the same resistive-screen filtering problem

by Lawrie and Afzal (now ref. [31]), "Higher-mode filtering: optimum attenuation in a continuum of exceptional points", Proc. R. Soc. A 2026;482(2340):20250811, doi:10.1098/rspa.2025.0811. Because that study is a frequency-domain analysis rather than a time-domain one, we have also rewritten every passage that referred to the preprint so that the description matches what the published paper contains: it is introduced as an analysis of optimum higher-mode attenuation by resistive wire-mesh perforates in a rigid duct, it is cited as a resistive-screen antecedent rather than as a time-domain one, and the wavepacket–Fourier reconstruction is now attributed solely to ref. [30].

Comment 5. *References are not numbered in order of first appearance ([25] is cited before [16], [28] before [19], etc.); renumber according to journal style.*

Response. The entire reference list has been renumbered in strict order of first appearance and reformatted to Vancouver style as required by PLOS ONE. The main moves relative to the previous numbering: old [25]–[28] (Nawaz et al. 2015; Afzal–Shafique 2020; Afzal et al. 2021; Afzal et al. 2022) now appear as [16], [22], [23], [20] at their first citations inside the methodology review; old [16]–[21] shift accordingly; the benchmark and time-domain papers close the original list as [29]–[30]; the unpublished preprint that was old [24] has been removed and replaced at [31] by a published article (see Comment 4 below); and two references added in response to Reviewer 1 (Delany–Bazley 1970; Kirby–Cummings 1999) enter as [32]–[33] at their first citations in the formulation section. Because virtually every entry changed number, the reference list is attached without change-marking in the marked-up copy, as noted above.

Comment 6. *Please state the wavenumber-grid parameters used in Eq. (23) (range and N_k) and note that truncating Eq. (21) at $k = 0$ is immaterial here because k_0 is large compared with σ_k.*

Response. Both grids are now stated after new Eq (26): the field snapshots use 40 wavenumber points spanning 2.5 rad/m on either side of the reference carrier of 9.15 rad/m, with a spectral bandwidth of 1.0 rad/m; the transient-energy studies use 80 points spanning three and a half bandwidths on either side of the carrier, with a bandwidth of 2.5 rad/m. We also quantified the reviewer's observation rather than merely asserting it: the fraction of the Gaussian spectral weight lying below zero wavenumber is at most 9.5×10^{-4} for the lowest carrier–bandwidth combination used (300 Hz carrier with the wider bandwidth) and below 1.2×10^{-7} in every other case, so the truncation is indeed immaterial; the revised text states these bounds. In stating the per-family parameters we also replaced the previous phrase "the bandwidth 2.5 rad/m is used throughout", which was accurate for the transient-energy family only, by the precise per-family statement.

Comment 7. *The space–time origin of the incident wavepacket (where the envelope centre sits at $t = 0$) should be stated in the text so the quoted snapshot times of the time-domain reconstruction are self-contained.*

Response. Stated in three places. In the time-reconstruction section: because of the phase convention chosen for the incident wave, the envelope centre travels at the sound speed and crosses the left membrane plane exactly at time zero, so negative times show the approach and positive times the transit and ring-down. The snapshot discussion and the caption of Fig 7 repeat the statement, and the description of panel (c) was sharpened to match it ("the instant at which the envelope centre sits on the left membrane plane – the leading half of the pulse already occupies the porous core").

Comment 8. *The convergence study fixes $M = N + 8$ without justification; briefly comment on the choice of the Galerkin-to-acoustic truncation ratio, since convergence of such schemes is known*

to depend on it.

Response. A justification and a quantitative sensitivity statement now follow the convergence discussion. Qualitatively: the sine basis must resolve the trace of the truncated cosine expansion on the membrane, and the closed-form projection couples each sine mode to cosine modes of every index of opposite parity, so a modest surplus of Galerkin modes over acoustic modes is the natural choice. Quantitatively: at the reference acoustic truncation of 16 modes, varying the surplus between 2 and 14 changes each of the three power fractions by less than 3×10^{-5} and leaves the membrane-velocity residual between 4.0×10^{-4} and 9.2×10^{-4}, with no plateau or degradation – so the specific offset is uncritical, and both choices used in the paper (a surplus of 8 in the convergence study and of 6 at the reference truncation) sit inside the insensitive range. The revised text says exactly this.

Comment 9. *For a membrane governed by the second-order equation (8) only the displacement can be prescribed, so "clamped" here coincides with fixed edges; a one-line remark would avoid confusion with plate-type clamping.*

Response. Added immediately after new Eq (9): because the membrane equation is second order in the transverse coordinate, only the edge displacement can be prescribed; the term "clamped" here therefore coincides with fixed (zero-displacement) edges, and no edge-slope condition arises – in contrast to fourth-order plate models, in which a clamped edge additionally constrains the rotation.

Comment 10. *Given the HVAC/silencer motivation and the cited mean-flow literature [14, 15], add a sentence acknowledging that mean flow, membrane damping and bending stiffness are neglected, and indicate the regime in which this is acceptable.*

Response. Added as the closing paragraph of the formulation section: the model neglects mean flow, membrane damping and membrane bending stiffness; dissipative-silencer analyses that include mean flow [14,15] find convective corrections to the axial wavenumbers of the order of the Mach number, so the results are the leading-order description for Mach numbers up to a few percent (the regime of ventilation and building-services ducts); membrane damping would round and lower the hybrid-resonance absorption peaks without displacing them appreciably; and bending stiffness is negligible for thin, tension-dominated films at the transverse wavelengths resolved by the Galerkin basis.

We hope the revised manuscript now meets the standards of PLOS ONE, and we remain at the disposal of the editor and the reviewers for any further clarification.
