# Supplementary material for: Computational time-dependent acoustic scattering in a rectangular membrane–porous waveguide
Source: PLoS One. 2026 Sep 30;21(9):e0358579. doi: 10.1371/journal.pone.0358579 (PMC13626333; doi:10.1371/journal.pone.0358579)
Supplement: Supplementary file 2 [file pone.0358579.s002.docx]

The manuscript combines a mode-matching/Galerkin formulation with a wavepacket–Fourier time reconstruction for a membrane–porous rectangular filter. The topic is relevant, the formulation is largely sound and the presentation is generally clear, but the central validation claim is logically circular as written, key material data are missing for reproducibility, and the novelty relative to closely related work needs sharpening. The figures are appropriate as they stand and no changes to them are requested; my comments concern the text and the analysis. I therefore recommend major revision.

1. In Eq. (24) the absorbed fraction is defined as E_abs_ := 1 − E_r_ − E_t_, so identity (25) holds by construction; reporting |E_r_ + E_t_ + E_abs_ − 1| below 10^−15^ in Sec. 6 therefore validates nothing. Please compute the absorbed power independently (dissipation integral over the porous core plus membrane input power) and quote the resulting residual in the text, or at least verify E_r_ + E_t_ = 1 in the lossless limit (real Γ, τ).
2. In Eq. (4), σ = A_1_ρ^A2^ is presented as the flow resistivity with ρ declared as the air density; the standard empirical relation uses the bulk density of the fibrous material, a different quantity. Please correct the symbol, and report the numerical values of A_1_, A_2_, a_1_–a_8_ and the resulting σ actually used.
3. The frequency sweeps extend to 10–12 kHz for the higher-mode studies. Please state the range of ξ = fρ/σ realized and confirm it stays within the accepted validity window of the Delany–Bazley correlations.
4. The passage from Eq. (8) to the Galerkin systems (18)–(19) is very compressed; a short appendix with the intermediate algebra, including the orientation convention of the pressure jump at the right membrane, is needed so signs and coefficients can be verified.
5. Since Ref. [24] already applies the same wavepacket–Fourier reconstruction to a rectangular chamber, the introduction should state more precisely what is methodologically new here beyond replacing resistive sheets by membranes plus a porous core (e.g., the clamped-sine Galerkin coupling and the closed-form R_mn_).
