# Supplementary material for: Computational time-dependent acoustic scattering in a rectangular membrane–porous waveguide
Source: PLoS One. 2026 Sep 30;21(9):e0358579. doi: 10.1371/journal.pone.0358579 (PMC13626333; doi:10.1371/journal.pone.0358579)
Supplement: Supplementary file 3 [file pone.0358579.s003.docx]

The formulation is consistent, the pointwise matching reconstruction and the truncation convergence study are convincing, and the parametric and time-domain results are clearly interpreted. The figures adequately support the text and no changes or additions to them are requested. A number of small corrections and clarifications listed below, all confined to the text, should be addressed before acceptance.

1. The units of W_a_(t) in Eq. (28) are stated as m^4^/s^2^, but with φ in m^2^/s the double integral yields m^6^/s^2^; the stated unit is that of the integrand only. Please correct.
2. Since φ(x, y, t) in Eq. (28) is real-valued, the modulus signs are redundant; also clarify in the text that |φ|^2^ is a field-intensity proxy rather than the acoustic energy density, or use a density based on |∇φ|^2^ and (∂_t_φ)^2^/c^2^.
3. The final sentence of the abstract ends with “an algebraic conservation identity satisfied”, which is grammatically incomplete; please rephrase.
4. Reference [24] is an unpublished preprint cited without year or identifier; complete the bibliographic data or explicitly mark it as unpublished.
5. References are not numbered in order of first appearance ([25] is cited before [16], [28] before [19], etc.); renumber according to journal style.
6. Please state the wavenumber-grid parameters used in Eq. (23) (range and N_k_) and note that truncating Eq. (21) at k = 0 is immaterial here because k_0_ is large compared with σ_k_.
7. The space–time origin of the incident wavepacket (where the envelope centre sits at t = 0) should be stated in the text so the quoted snapshot times of the time-domain reconstruction are self-contained.
8. The convergence study fixes M = N + 8 without justification; briefly comment on the choice of the Galerkin-to-acoustic truncation ratio, since convergence of such schemes is known to depend on it.
9. For a membrane governed by the second-order equation (8) only the displacement can be prescribed, so “clamped” here coincides with fixed edges; a one-line remark would avoid confusion with plate-type clamping.
10. Given the HVAC/silencer motivation and the cited mean-flow literature [14, 15], add a sentence acknowledging that mean flow, membrane damping and bending stiffness are neglected, and indicate the regime in which this is acceptable.
