# Supplementary material for: Computational time-dependent acoustic scattering in a rectangular membrane–porous waveguide
Source: PLoS One. 2026 Sep 30;21(9):e0358579. doi: 10.1371/journal.pone.0358579 (PMC13626333; doi:10.1371/journal.pone.0358579)
Supplement: Peer review history

# Peer review: Decision Letter 0

**DOI:** 10.1371/journal.pone.0358579.r001

**Authors:** Rab Nawaz

24 Jul 2026

Dear Dr. Safdar,

Thank you for submitting your manuscript to PLOS ONE. After careful consideration, we feel that it has merit but does not fully meet PLOS ONE’s publication criteria as it currently stands. Therefore, we invite you to submit a revised version of the manuscript that addresses the points raised during the review process.

Please submit your revised manuscript by Sep 22 2026 11:59PM. If you will need more time than this to complete your revisions, please reply to this message or contact the journal office at plosone@plos.org. When you're ready to submit your revision, log on to https://www.editorialmanager.com/pone/ and select the 'Submissions Needing Revision' folder to locate your manuscript file.

- A letter that responds to each point raised by the academic editor and reviewer(s). You should upload this letter as a separate file labeled 'Response to Reviewers'.
- A marked-up copy of your manuscript that highlights changes made to the original version. You should upload this as a separate file labeled 'Revised Manuscript with Track Changes'.
- An unmarked version of your revised paper without tracked changes. You should upload this as a separate file labeled 'Manuscript'.

If you would like to make changes to your financial disclosure, please include your updated statement in your cover letter. Guidelines for resubmitting your figure files are available below the reviewer comments at the end of this letter.

If applicable, we recommend that you deposit your laboratory protocols in protocols.io to enhance the reproducibility of your results. Protocols.io assigns your protocol its own identifier (DOI) so that it can be cited independently in the future. For instructions see: https://journals.plos.org/plosone/s/submission-guidelines#loc-laboratory-protocols. Additionally, PLOS ONE offers an option for publishing peer-reviewed Lab Protocol articles, which describe protocols hosted on protocols.io. Read more information on sharing protocols at https://plos.org/protocols?utm_medium=editorial-email&utm_source=authorletters&utm_campaign=protocols.

As the corresponding author, your ORCID iD is verified in the submission system and will appear in the published article. PLOS supports the use of ORCID, and we encourage all coauthors to register for an ORCID iD and use it as well. Please encourage your coauthors to verify their ORCID iD within the submission system before final acceptance, as unverified ORCID iDs will not appear in the published article. *Only*  the individual author can complete the verification step; PLOS staff *cannot*  verify ORCID iDs on behalf of authors.

We look forward to receiving your revised manuscript.

Kind regards,

Prof Dr Rab Nawaz

Academic Editor

PLOS One

Journal Requirements:

When submitting your revision, we need you to address these additional requirements.

1. Please ensure that your manuscript meets PLOS ONE's style requirements, including those for file naming. The PLOS ONE style templates can be found at

https://journals.plos.org/plosone/s/file?id=wjVg/PLOSOne_formatting_sample_main_body.pdf and

https://journals.plos.org/plosone/s/file?id=ba62/PLOSOne_formatting_sample_title_authors_affiliations.pdf

2. Please update your submission to use the PLOS LaTeX template. The template and more information on our requirements for LaTeX submissions can be found at http://journals.plos.org/plosone/s/latex.

3. If the reviewer comments include a recommendation to cite specific previously published works, please review and evaluate these publications to determine whether they are relevant and should be cited. There is no requirement to cite these works unless the editor has indicated otherwise.

[Note: HTML markup is below. Please do not edit.]

Reviewers' comments:

Reviewer's Responses to Questions

**Comments to the Author**

1. Is the manuscript technically sound, and do the data support the conclusions?

Reviewer #1: Yes

Reviewer #2: Yes

**********

2. Has the statistical analysis been performed appropriately and rigorously?

Reviewer #1: Yes

Reviewer #2: N/A

**********

3. Have the authors made all data underlying the findings in their manuscript fully available?

The PLOS Data policy

Reviewer #1: Yes

Reviewer #2: Yes

**********

4. Is the manuscript presented in an intelligible fashion and written in standard English?

Reviewer #1: Yes

Reviewer #2: Yes

**********

Reviewer #1: The manuscript combines a mode-matching/Galerkin formulation with a wavepacket–Fourier time reconstruction for a membrane–porous rectangular filter. The topic is relevant, the formulation is largely sound and the presentation is generally clear, but the central validation claim is logically circular as written, key material data are missing for reproducibility, and the novelty relative to closely related work needs sharpening. The figures are appropriate as they stand and no changes to them are requested; my comments concern the text and the analysis. I therefore recommend major revision.

1. In Eq. (24) the absorbed fraction is defined as Eabs := 1 − Er − Et, so identity (25) holds by construction; reporting |Er + Et + Eabs − 1| below 10−15 in Sec. 6 therefore validates nothing. Please compute the absorbed power independently (dissipation integral over the porous core plus membrane input power) and quote the resulting residual in the text, or at least verify Er + Et = 1 in the lossless limit (real Γ, τ).

2. In Eq. (4), σ = A1ρA2 is presented as the flow resistivity with ρ declared as the air density; the standard empirical relation uses the bulk density of the fibrous material, a different quantity. Please correct the symbol, and report the numerical values of A1, A2, a1–a8 and the resulting σ actually used.

3. The frequency sweeps extend to 10–12 kHz for the higher-mode studies. Please state the range of = fρ/σ realized and confirm it stays within the accepted validity window of the Delany–Bazley correlations.

4. The passage from Eq. (8) to the Galerkin systems (18)–(19) is very compressed; a short appendix with the intermediate algebra, including the orientation convention of the pressure jump at the right membrane, is needed so signs and coefficients can be verified.

5. Since Ref. [24] already applies the same wavepacket–Fourier reconstruction to a rectangular chamber, the introduction should state more precisely what is methodologically new here beyond replacing resistive sheets by membranes plus a porous core (e.g., the clamped-sine Galerkin coupling and the closed-form Rmn).

Reviewer #2: The formulation is consistent, the pointwise matching reconstruction and the truncation convergence study are convincing, and the parametric and time-domain results are clearly interpreted. The figures adequately support the text and no changes or additions to them are requested. A number of small corrections and clarifications listed below, all confined to the text, should be addressed before acceptance.

1. The units of Wa(t) in Eq. (28) are stated as m4/s2, but with ϕ in m2/s the double integral yields m6/s2; the stated unit is that of the integrand only. Please correct.

2. Since ϕ(x, y, t) in Eq. (28) is real-valued, the modulus signs are redundant; also clarify in the text that |ϕ|2 is a field-intensity proxy rather than the acoustic energy density, or use a density based on |∇ϕ|2 and (∂tϕ)2/c2.

3. The final sentence of the abstract ends with “an algebraic conservation identity satisfied”, which is grammatically incomplete; please rephrase.

4. Reference [24] is an unpublished preprint cited without year or identifier; complete the bibliographic data or explicitly mark it as unpublished.

5. References are not numbered in order of first appearance ([25] is cited before [16], [28] before [19], etc.); renumber according to journal style.

6. Please state the wavenumber-grid parameters used in Eq. (23) (range and Nk) and note that truncating Eq. (21) at k = 0 is immaterial here because k0 is large compared with σk.

7. The space–time origin of the incident wavepacket (where the envelope centre sits at t = 0) should be stated in the text so the quoted snapshot times of the time-domain reconstruction are self-contained.

8. The convergence study fixes M = N + 8 without justification; briefly comment on the choice of the Galerkin-to-acoustic truncation ratio, since convergence of such schemes is known to depend on it.

9. For a membrane governed by the second-order equation (8) only the displacement can be prescribed, so “clamped” here coincides with fixed edges; a one-line remark would avoid confusion with plate-type clamping.

10. Given the HVAC/silencer motivation and the cited mean-flow literature [14, 15], add a sentence acknowledging that mean flow, membrane damping and bending stiffness are neglected, and indicate the regime in which this is acceptable.

**********

what does this mean?). If published, this will include your full peer review and any attached files.

If you choose “no”, your identity will remain anonymous but your review may still be made public.

**Do you want your identity to be public for this peer review?**  For information about this choice, including consent withdrawal, please see our Privacy Policy

Reviewer #1: No

Reviewer #2: No

**********

[NOTE: If reviewer comments were submitted as an attachment file, they will be attached to this email and accessible via the submission site. Please log into your account, locate the manuscript record, and check for the action link "View Attachments". If this link does not appear, there are no attachment files.]

To ensure your figures meet our technical requirements, please review our figure guidelines: https://journals.plos.org/plosone/s/figures

You may also use PLOS’s free figure tool, NAAS, to help you prepare publication quality figures: https://journals.plos.org/plosone/s/figures#loc-tools-for-figure-preparation.

NAAS will assess whether your figures meet our technical requirements by comparing each figure against our figure specifications.

Attachment Submitted filename: reportplos.docx

Attachment Submitted filename: Comments (1).docx

# Author comment: Author response to Decision Letter 1

**DOI:** 10.1371/journal.pone.0358579.r002

25 Aug 2026

Reviewer 2

General comments. The formulation is consistent, the pointwise matching reconstruction and

the truncation convergence study are convincing, and the parametric and time-domain results are

clearly interpreted. The figures adequately support the text and no changes or additions to them

are requested. A number of small corrections and clarifications listed below, all confined to the text,

should be addressed before acceptance.

Response. We thank the reviewer for the positive assessment and for ten precise, constructive

corrections; all ten are implemented, and no figure has been altered.

Comment 1. The units of Wa(t) in Eq. (28) are stated as m4/s2, but with φ in m2/s the double

integral yields m6/s2; the stated unit is that of the integrand only. Please correct.

Response. Correct – the stated unit was that of the integrand. New Eq (33) now carries m6/s2,

and the following sentence spells out the bookkeeping: with the potential in m2/s the squared

integrand carries m4/s2, and the double integral over the core area contributes the remaining m2.

Comment 2. Since φ(x, y, t) in Eq. (28) is real-valued, the modulus signs are redundant; also

clarify in the text that |φ|2 is a field-intensity proxy rather than the acoustic energy density, or use

a density based on |∇φ|2 and (∂tφ)2/c2.

Response. Both done, following the reviewer’s first option. The integrand of new Eq (33) is now

written as the plain square of the real-valued time-domain field, with an explicit remark that no

modulus is needed. The text then states that the quantity is “a field-intensity proxy for the energy

content of the core, not the acoustic energy density itself” – the latter would combine the squared

field gradient with the squared time derivative of the field – notes that it is likewise distinct from

the steady-state absorbed fraction, and adds that for the narrow-band wavepackets used here the

two energy-like measures are monotonically related, which is what justifies using the proxy for the

comparative transit studies.

Comment 3. The final sentence of the abstract ends with “an algebraic conservation identity

satisfied”, which is grammatically incomplete; please rephrase.

Response. Rephrased – and, in view of Reviewer 1’s first comment, the sentence now describes

the strengthened validation rather than the circular identity. It reads: “The block linear system is

validated by point-wise reconstruction of pressure and velocity continuity at every matching plane,

by a truncation convergence study, and by two independent energy checks: the absorbed power

fraction is recomputed from a dissipation audit of the power fluxes entering and leaving the porous

core, and the lossless limit of the porous model is shown to return the incident power in full.”

Comment 4. Reference [24] is an unpublished preprint cited without year or identifier; complete

the bibliographic data or explicitly mark it as unpublished.

Response. We have gone further than marking it. The unpublished preprint has been removed

from the manuscript altogether, so that every entry in the reference list is now a published, citable

source. In its place we cite the peer-reviewed treatment of the same resistive-screen filtering problem

4by Lawrie and Afzal (now ref. [31]), “Higher-mode filtering: optimum attenuation in a continuum

of exceptional points”, Proc. R. Soc. A 2026;482(2340):20250811, doi:10.1098/rspa.2025.0811. Because that study is a frequency-domain analysis rather than a time-domain one, we have also

rewritten every passage that referred to the preprint so that the description matches what the

published paper contains: it is introduced as an analysis of optimum higher-mode attenuation by

resistive wire-mesh perforates in a rigid duct, it is cited as a resistive-screen antecedent rather

than as a time-domain one, and the wavepacket–Fourier reconstruction is now attributed solely to

ref. [30].

Comment 5. References are not numbered in order of first appearance ([25] is cited before [16],

[28] before [19], etc.); renumber according to journal style.

Response. The entire reference list has been renumbered in strict order of first appearance and

reformatted to Vancouver style as required by PLOS ONE. The main moves relative to the previous

numbering: old [25]–[28] (Nawaz et al. 2015; Afzal–Shafique 2020; Afzal et al. 2021; Afzal et al. 2022)

now appear as [16], [22], [23], [20] at their first citations inside the methodology review; old [16]–[21]

shift accordingly; the benchmark and time-domain papers close the original list as [29]–[30]; the

unpublished preprint that was old [24] has been removed and replaced at [31] by a published article

(see Comment 4 below); and two references added in response to Reviewer 1 (Delany–Bazley 1970;

Kirby–Cummings 1999) enter as [32]–[33] at their first citations in the formulation section. Because

virtually every entry changed number, the reference list is attached without change-marking in the

marked-up copy, as noted above.

Comment 6. Please state the wavenumber-grid parameters used in Eq. (23) (range and Nk) and

note that truncating Eq. (21) at k = 0 is immaterial here because k0 is large compared with σk.

Response. Both grids are now stated after new Eq (26): the field snapshots use 40 wavenumber

points spanning 2.5 rad/m on either side of the reference carrier of 9.15 rad/m, with a spectral

bandwidth of 1.0 rad/m; the transient-energy studies use 80 points spanning three and a half

bandwidths on either side of the carrier, with a bandwidth of 2.5 rad/m. We also quantified the

reviewer’s observation rather than merely asserting it: the fraction of the Gaussian spectral weight

lying below zero wavenumber is at most 9.5 × 10−4 for the lowest carrier–bandwidth combination

used (300 Hz carrier with the wider bandwidth) and below 1.2 × 10−7 in every other case, so the

truncation is indeed immaterial; the revised text states these bounds. In stating the per-family

parameters we also replaced the previous phrase “the bandwidth 2.5 rad/m is used throughout”,

which was accurate for the transient-energy family only, by the precise per-family statement.

Comment 7. The space–time origin of the incident wavepacket (where the envelope centre sits at

t = 0) should be stated in the text so the quoted snapshot times of the time-domain reconstruction

are self-contained.

Response. Stated in three places. In the time-reconstruction section: because of the phase

convention chosen for the incident wave, the envelope centre travels at the sound speed and crosses

the left membrane plane exactly at time zero, so negative times show the approach and positive

times the transit and ring-down. The snapshot discussion and the caption of Fig 7 repeat the

statement, and the description of panel (c) was sharpened to match it (“the instant at which the

envelope centre sits on the left membrane plane – the leading half of the pulse already occupies the

porous core”).

Comment 8. The convergence study fixes M = N + 8 without justification; briefly comment on

the choice of the Galerkin-to-acoustic truncation ratio, since convergence of such schemes is known

5to depend on it.

Response. A justification and a quantitative sensitivity statement now follow the convergence

discussion. Qualitatively: the sine basis must resolve the trace of the truncated cosine expansion

on the membrane, and the closed-form projection couples each sine mode to cosine modes of every

index of opposite parity, so a modest surplus of Galerkin modes over acoustic modes is the natural

choice. Quantitatively: at the reference acoustic truncation of 16 modes, varying the surplus

between 2 and 14 changes each of the three power fractions by less than 3 × 10−5 and leaves the

membrane-velocity residual between 4.0×10−4 and 9.2×10−4, with no plateau or degradation – so

the specific offset is uncritical, and both choices used in the paper (a surplus of 8 in the convergence

study and of 6 at the reference truncation) sit inside the insensitive range. The revised text says

exactly this.

Comment 9. For a membrane governed by the second-order equation (8) only the displacement

can be prescribed, so “clamped” here coincides with fixed edges; a one-line remark would avoid

confusion with plate-type clamping.

Response. Added immediately after new Eq (9): because the membrane equation is second order

in the transverse coordinate, only the edge displacement can be prescribed; the term “clamped”

here therefore coincides with fixed (zero-displacement) edges, and no edge-slope condition arises

– in contrast to fourth-order plate models, in which a clamped edge additionally constrains the

rotation.

Comment 10. Given the HVAC/silencer motivation and the cited mean-flow literature [14, 15],

add a sentence acknowledging that mean flow, membrane damping and bending stiffness are neglected, and indicate the regime in which this is acceptable.

Response. Added as the closing paragraph of the formulation section: the model neglects mean

flow, membrane damping and membrane bending stiffness; dissipative-silencer analyses that include

mean flow [14,15] find convective corrections to the axial wavenumbers of the order of the Mach

number, so the results are the leading-order description for Mach numbers up to a few percent (the

regime of ventilation and building-services ducts); membrane damping would round and lower the

hybrid-resonance absorption peaks without displacing them appreciably; and bending stiffness is

negligible for thin, tension-dominated films at the transverse wavelengths resolved by the Galerkin

basis.

We hope the revised manuscript now meets the standards of PLOS ONE, and we remain at the

disposal of the editor and the reviewers for any further clarification

Attachment Submitted filename: Response_to_Reviewers.pdf

# Peer review: Decision Letter 1

**DOI:** 10.1371/journal.pone.0358579.r003

**Authors:** Rab Nawaz

2 Sep 2026

Computational time-dependent acoustic scattering in a rectangular membrane--porous waveguide

PONE-D-26-30398R1

Dear Dr Muhammad Safdar,

We’re pleased to inform you that your manuscript has been judged scientifically suitable for publication and will be formally accepted for publication once it meets all outstanding technical requirements.

Within one week, you’ll receive an e-mail detailing the required amendments. When these have been addressed, you’ll receive a formal acceptance letter and your manuscript will be scheduled for publication.

An invoice will be generated when your article is formally accepted. Please note, if your institution has a publishing partnership with PLOS and your article meets the relevant criteria, all or part of your publication costs will be covered. Please make sure your user information is up-to-date by logging into Editorial Manager at Editorial Manager® and clicking the ‘Update My Information' link at the top of the page. For questions related to billing, please contact billing support.

If your institution or institutions have a press office, please notify them about your upcoming paper to help maximize its impact. If they’ll be preparing press materials, please inform our press team as soon as possible -- no later than 48 hours after receiving the formal acceptance. Your manuscript will remain under strict press embargo until 2 pm Eastern Time on the date of publication. For more information, please contact onepress@plos.org.

Kind regards,

Academic Editor

PLOS One

Additional Editor Comments (optional):

Reviewers' comments:

Reviewer's Responses to Questions

**Comments to the Author**

Reviewer #1: All comments have been addressed

Reviewer #2: All comments have been addressed

Reviewer #3: All comments have been addressed

**********

2. Is the manuscript technically sound, and do the data support the conclusions?

Reviewer #1: Yes

Reviewer #2: Yes

Reviewer #3: Yes

**********

3. Has the statistical analysis been performed appropriately and rigorously?

Reviewer #1: Yes

Reviewer #2: (No Response)

Reviewer #3: Yes

**********

4. Have the authors made all data underlying the findings in their manuscript fully available?

The PLOS Data policy

Reviewer #1: Yes

Reviewer #2: Yes

Reviewer #3: Yes

**********

5. Is the manuscript presented in an intelligible fashion and written in standard English?

Reviewer #1: Yes

Reviewer #2: Yes

Reviewer #3: Yes

**********

Reviewer #1: (No Response)

Reviewer #2: The revised manuscript has adequately addressed all the points raised during the first round of review. I am pleased to recommend its acceptance in its present form without further revisions.

Reviewer #3: (No Response)

**********

what does this mean?). If published, this will include your full peer review and any attached files.

If you choose “no”, your identity will remain anonymous but your review may still be made public.

**Do you want your identity to be public for this peer review?**  For information about this choice, including consent withdrawal, please see our Privacy Policy

Reviewer #1: No

Reviewer #2: No

Reviewer #3: No

**********

# Editor report: Acceptance letter

**DOI:** 10.1371/journal.pone.0358579.r004

**Authors:** Rab Nawaz

PONE-D-26-30398R1

PLOS One

Dear Dr. Safdar,

I'm pleased to inform you that your manuscript has been deemed suitable for publication in PLOS One. Congratulations! Your manuscript is now being handed over to our production team.

At this stage, our production department will prepare your paper for publication. This includes ensuring the following:

* All references, tables, and figures are properly cited

* All relevant supporting information is included in the manuscript submission,

* There are no issues that prevent the paper from being properly typeset

You will receive further instructions from the production team, including instructions on how to review your proof when it is ready. Please keep in mind that we are working through a large volume of accepted articles, so please give us a few days to review your paper and let you know the next and final steps.

Lastly, if your institution or institutions have a press office, please let them know about your upcoming paper now to help maximize its impact. If they'll be preparing press materials, please inform our press team within the next 48 hours. Your manuscript will remain under strict press embargo until 2 pm Eastern Time on the date of publication. For more information, please contact onepress@plos.org.

You will receive an invoice from PLOS for your publication fee after your manuscript has reached the completed accept phase. If you receive an email requesting payment before acceptance or for any other service, this may be a phishing scheme. Learn how to identify phishing emails and protect your accounts at https://explore.plos.org/phishing.

If we can help with anything else, please email us at customercare@plos.org.

Thank you for submitting your work to PLOS One and supporting open access.

Kind regards,

PLOS One Editorial Office Staff

on behalf of

Dr. Rab Nawaz

Academic Editor

PLOS One
